# Multifactorial Perspective for Greening Solid‐Phase Peptide Synthesis: Rigid Polyacrylate Macroporous Resin in Combination with Green Solvents

**DOI:** 10.1002/cssc.70591

**Published:** 2026-04-14

**Authors:** Sikabwe Noki, Ashish Kumar, Cheng Zhang, Alesandra Basso, Simona Serban, Xiaokang Kou, Yanjun Li, Anamika Sharma, Beatriz G. de la Torre, Fernando Albericio

**Affiliations:** ^1^ Peptide Science Laboratory School of Chemistry and Physics University of KwaZulu‐Natal Durban South Africa; ^2^ School of Laboratory Medicine and Medical Sciences College of Health Sciences University of KwaZulu‐Natal Durban South Africa; ^3^ Sunresin New Materials Xi’an China; ^4^ Department of Inorganic and Organic Chemistry University of Barcelona Barcelona Spain

**Keywords:** green solvent, polyacrylate macroporous resin, SPPS, sustainability

## Abstract

Until now, efforts to 'green' solid‐phase peptide synthesis (SPPS) have largely focused on identifying a single solvent capable of replacing dimethylformamide, while relying exclusively on polystyrene‐based resins. This approach has proven both challenging and limiting. Here, we propose that the most effective strategy for incorporating greener solvents into SPPS is to identify resin solvent combinations that can simultaneously support the two key reactions in peptide synthesis: coupling and Fmoc deprotection. When a relatively rigid resin is used, resin swelling is no longer a critical parameter in SPPS. This, in turn, enables the use of solvents that do not need to swell the resin, greatly expanding the range of greener solvent options that can be considered. This concept is exemplified by a novel polyacrylate‐based rigid macroporous resin, which has enabled the SPPS synthesis of small‐ to medium‐sized peptides using EtOAc, TEP, 2‐MeTHF, acetone, and MeCN as single solvents.

## Graphical abstract

A rigid, non‐swelling polyacrylate resin enables greener solid‐phase peptide synthesis by allowing various green solvents during the peptide synthesis and significantly reduced process mass intensity without compromising peptide synthesis efficiency/purity.

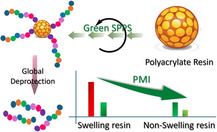

## 1 Introduction

Since the first synthesis of oxytocin by Nobel Laureate Vincent du Vigneaud, the role of peptides in science has undergone a profound transformation [1]. In the early days, peptides typically small in size were regarded merely as biochemical tools. However, advances in synthetic methodologies have elevated peptides to the status of blockbuster drugs, with multi‐ton production now feasible for sequences exceeding 40 residues [2, 3, 4].

A pivotal milestone was the development of solid‐phase peptide synthesis (SPPS) by Nobel Laureate R. Bruce Merrifield [5]. In its early years, however, SPPS faced scepticism regarding its ability to produce active pharmaceutical ingredients that met the stringent standards of regulatory authorities [6]. This doubt was dispelled at the beginning of the 21st century with the market launch of T‐20 (Enfuvirtide) for HIV‐1 treatment [7]. More recently, the success of tirzepatide (Mounjaro) for metabolic diseases has further demonstrated SPPS’ potential for large‐scale peptide production [8].

However, tirzepatide's success has also highlighted one of SPPS’ major sustainability challenges: solvent consumption. A single synthetic cycle may require at least ten washing steps, each using roughly ten volumes of solvent relative to the solid support (mL/g). Simple calculations involving resin weight, peptide length, and solvent requirements reveal that large‐scale manufacturing can consume tons of solvent per campaign. This reality has renewed interest in liquid‐phase peptide synthesis (LPPS) [9]. In LPPS, the resin is replaced by a lipophilic tag, and synthesis proceeds continuously, with excess reagents and byproducts removed via straightforward liquid–liquid extractions or precipitations. As in SPPS, intermediates are not purified between steps. LPPS requires significantly less solvent and uses fewer reagent excesses while maintaining comparable cycle times. Within SPPS itself, another strategy to reduce solvent usage involves minimising washing steps, for example, by adding the deprotection reagent immediately after coupling, omitting the intermediate wash, and washing only after deprotection [10, 11].

In SPPS, the choice of resin is critical for efficient synthesis. The most common support is polystyrene (PS) cross‐linked with divinylbenzene (DVB) (PS‐DVB) [12]. Resin performance depends strongly on its swelling capacity in the chosen solvent, which initially restricted SPPS to dichloromethane (DCM) and later to N, N‐dimethylformamide (DMF), both of which swell PS‐DVB effectively. Many improvements to PS‐DVB aimed to enhance swelling in DMF, first through grafting polyethylene glycol (PEG) [13] onto PS and later through the development of fully PEG‐based resins [14, 15, 16]. PEG resins generally outperform PS resins in terms of synthetic efficiency but come with sustainability drawbacks: they require even larger solvent volumes because of their higher swelling, and they typically offer lower loading capacities due to the increased mass of PEG‐PS resins. Fully PEG‐based resins, such as PEGA and ChemMatrix, represent the high‐swelling extreme [14, 15, 16]. While these resins produce peptides of excellent purity often superior to PEG‐PS and PS‐DVB, they demand even more solvent, and peptide recovery from them is frequently poor [17]. Moreover, several of these resins are no longer commercially available.

Here, we propose the use of rigid, non‐swelling resins with two major advantages. First, they substantially reduce solvent consumption, since the ‘volume’ occupied by a non‐swelling resin is much smaller than that of a swelling resin, requiring less solvent to fully cover the solid support. Second, these non‐swelling resins enable the use of alternative and predominantly greener solvents that are incompatible with traditional swelling resins such as PS‐DVB.

Only a few examples of non‐swelling or rigid resins exist in the literature. Macroporous polystyrene resins, similar to those used in ion‐exchange chromatography, have been used for the synthesis of small peptides [18, 19, 20]. Controlled pore glass, commonly employed for oligonucleotide synthesis, and silica supports have also been used occasionally for the solid‐phase synthesis of small peptides [20, 21, 22]. One of us proposed a similar resin to be used with DMF as a solvent [23]. Recently, Biondi and co‐workers proposed a novel polymeric mesoporous support (pDVB), with the same idea as us: the solid support should not be affected by the nature of the solvent [24]. Finally, our group demonstrated that small‐ to medium‐sized peptides can be synthesized using functionalized silica (SiliCycle), previously used as a scavenger for metals and genotoxic impurities, as well as for peptide purification and reagent/catalyst immobilization [25]. Use of SiliCycle support significantly reduced solvent consumption during washings.

In the present work, we propose an organic polymeric support based on methacrylate copolymers, formed from a functional monomer and a cross‐linker, for use in SPPS. The polyacrylate resin was copolymerized using 85% (mol/mol) GMA and 15% (mol/mol) DVB, with pore size of 30 nm, a typical specific surface area of 130 m^2^/g, and particle size 75–150 μm. Two versions of the solid support were prepared using a proprietary method, differing in the length of the spacer either two or six carbons between the core and the linker [Seplife PA‐C2 (short spacer) and Seplife PA‐C6 (long spacer) resins].

## 2 Result and Discussion

Using this rigid polyacrylate‐based macroporous resin serves two primary purposes. First, it enables SPPS to be carried out in green solvents that are typically incompatible with conventional polystyrene (PS‐DVB) resins, which require significant swelling to function effectively. Second, it permits the use of smaller solvent volumes for washing and, when possible, even during coupling, exploiting higher reagent concentrations to accelerate reaction kinetics. To support this approach, the solubility of Fmoc‐amino acid building blocks in a range of solvents was first assessed.

### 2.1 Solubility

A solubility analysis was conducted for each Fmoc‐amino acid. Solutions at 0.2 and 0.6 M were prepared by dissolving the test amino acid in 0.5 mL of the designated solvent. The solvents examined were dimethylformamide (DMF), triethyl phosphate (TEP), acetonitrile (MeCN), ethyl acetate (EtOAc), acetone, 2‐methyl tetrahydrofuran (2‐MeTHF), and tetrahydrofuran (THF) (Table 1).

**TABLE 1:** Solubility study of Fmoc‐protected amino acids in selected solvents at 0.2 M and 0.6 M concentration.^a^

	DMF		TEP		MeCN		EtOAc		Acetone		2‐MeTHF		THF	
	*A*	*B*	*A*	*B*	*A*	*B*	*A*	*B*	*A*	*B*	*A*	*B*	*A*	*B*
Fmoc‐Leu‐OH														
Fmoc‐Phe‐OH														
Fmoc‐Gly‐OH														
Fmoc‐Tyr(*t*Bu)‐OH														
Fmoc‐Ser(*t*Bu)‐OH														
Fmoc‐Pro‐OH														
Fmoc‐Ala‐OH														
Fmoc‐Val‐OH														
Fmoc‐Lys(Boc)‐OH														
Fmoc‐Trp(Boc)‐OH														
Fmoc‐Arg(Pbf)‐OH														
Fmoc‐His(Trt)‐OH														
Fmoc‐Glu(O*t*Bu)‐OH														
Fmoc‐Ile‐OH														
Fmoc‐Asp(OtBu)‐OH														

*Note:* A = 0.2 M; B = 0.6 M; soluble (Green); partially soluble (Yellow); insoluble (Red).

a Fmoc‐Gln/Asn(Trt)‐OH were not analysed, because they are not part of the sequence synthesized. However, poor solubility of these two Fmoc‐amino acids is envisaged in this collection of solvents.

At 0.2 M, Fmoc‐Leu‐OH, Fmoc‐Ser(tBu)‐OH, Fmoc‐Pro‐OH, Fmoc‐Ala‐OH, Fmoc‐Val‐OH, and Fmoc‐Trp(Boc)‐OH were fully soluble in DMF, TEP, acetone, 2‐MeTHF, and THF. MeCN and EtOAc were consistently the least effective solvents and often required activation with DIC in the presence of OxymaPure, along with heating to 45°C, to achieve dissolution. Similar trends were observed at 0.6 M: DMF, TEP, 2‐MeTHF, and THF continued to dissolve most amino acids completely, whereas MeCN and EtOAc typically failed to dissolve the majority of Fmoc‐Aaa(PG)‐OH building blocks even after DIC addition.

For aromatic amino acids such as Fmoc‐Phe‐OH and Fmoc‐Tyr(tBu)‐OH, partial or complete insolubility was observed in MeCN, EtOAc, and sometimes acetone at both concentrations. However, DIC/OxymaPure addition followed by heating enabled dissolution in all cases at 0.2 M and in most cases at 0.6 M. Fmoc‐Gly‐OH showed the same behaviour, remaining insoluble in MeCN and EtOAc at both concentrations but dissolving after DIC activation and heating.

Other amino acids showed more pronounced solvent sensitivity. Fmoc‐Lys(Boc)‐OH was insoluble in MeCN and EtOAc even at 0.2 M and required DIC/Oxyma activation and 45°C heating to dissolve fully. Fmoc‐Arg(Pbf)‐OH and Fmoc‐His(Trt)‐OH exhibited limited solubility in all solvents except DMF, with partial solubility in TEP and THF at 0.2 M and complete insolubility in MeCN, EtOAc, acetone, and 2‐MeTHF at both concentrations. These amino acids consistently required DIC/OxymaPure activation and heating for dissolution. Acidic amino acids behaved more favourably: Fmoc‐Glu(OtBu)‐OH was completely soluble in all solvents at both concentrations, whereas Fmoc‐Asp(OtBu)‐OH and Fmoc‐Ile‐OH were insoluble in MeCN and EtOAc at both concentrations but dissolved after DIC/OxymaPure activation and heating. Notably, Fmoc‐Val‐OH and Fmoc‐Leu‐OH showed concentration‐independent insolubility in MeCN and EtOAc, although coupling reactions still proceeded despite incomplete dissolution.

Overall, these results reveal that solvent identity exerts a far greater influence on solubility than concentration. DMF, TEP, 2‐MeTHF, and THF consistently supported broad solubilization across diverse amino acids, whereas MeCN and EtOAc remained poor solvents for Fmoc‐Aaa(PG)‐OH. Although DIC/OxymaPure activation combined with mild heating can overcome solubility limitations in many cases, the data collectively highlight that solvent choice—not concentration—is the dominant factor governing solubility. Importantly, greener solvents such as TEP and 2‐MeTHF emerge as robust alternatives to DMF, providing high solubility across a wide range of amino acids (Figures S1–S16).

### 2.2 Swelling

**FIGURE 1:**
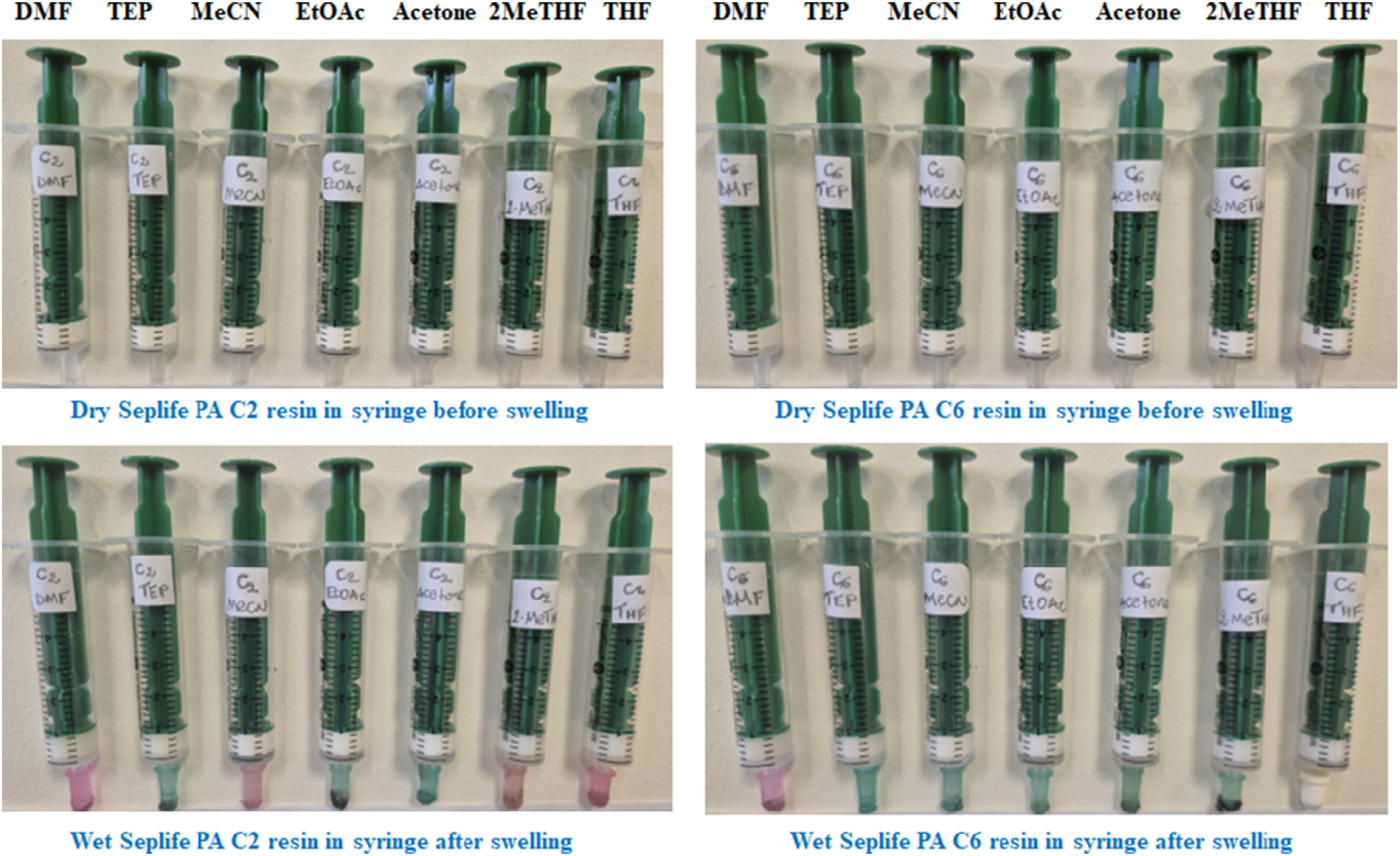
Swelling study of Seplife PA‐C2/C6 resins in DMF, TEP, MeCN, EtOAc, Acetone, 2‐MeTHF, and THF after 30 min.

Unlike conventional polystyrene‐based resins (PS‐DVB), the Seplife PA‐C2 and PA‐C6 resins do not swell in any common organic solvent. To confirm this lack of swelling, 200 mg of each resin was suspended in DMF, TEP, MeCN, EtOAc, acetone, 2‐MeTHF, and THF, these solvents known to swell PS‐DVB efficiently. No observable swelling was detected in either case (Figure 1), confirming that the Seplife PA‐C2 and PA‐C6 resins retain a rigid structure without solvent‐induced expansion.

**FIGURE 2:**
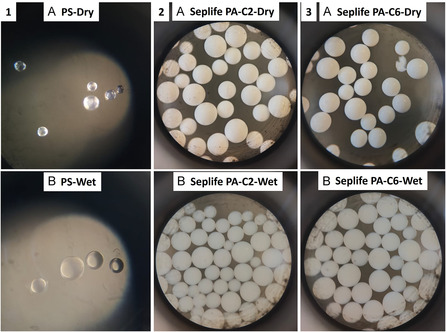
Microscopic study of 1) polystyrene (PS) resin; 2) Seplife PA‐C2 resin; and 3) Seplife PA‐C6 resin: (A) all resins in dry form; (B) all resins in DMF.

Figure 2 contains the microscopic images of the Seplife PA‐C2/C6 resins. In addition, a comparison has been made using polystyrene resin. It is well known that PS resin possesses swelling properties (Figure 2‐1A PS when dry and Figure 2‐1B **PS** when swelled in DMF). Upon comparison with Seplife PA‐C2 or Seplife PA‐C6 resins, they, on the contrary, do not show any swelling properties as shown in Figure 2.

### 2.3 Fmoc‐RinkAmide Linker Incorporation in Seplife PA‐C2/C6 Resins

Fmoc‐RinkAmide linker was incorporating into Seplife PA‐C2/C6 resins on 0.1‐mmol scale using DIC and OxymaPure [Fmoc‐RinkAmide linker‐DIC‐OxymaPure (1:1:1), 5 eq.] in DMF for 1h at room temperature (RT). The non‐reacted amino groups of the resin were subsequently capped with acetic anhydride (Ac_2_O) and *N*,*N*,diisopropylethylamine (DIPEA) (5 eq each) for 30 min (min). The loading capacity was determined by UV–VIS spectroscopy in the range of 260–340 nm of the dibenzofulvene adduct formed after removal of the Fmoc with piperidine [26]. The experimental loading after incorporation of the Fmoc‐RinkAmide linker was 0.40 mmol/g for C2 resin and 0.31 mmol/g for the C6 resin, compared with theoretical loading 0.63 and 0.57 mmol/g respectively.

### 2.4 Application of Seplife PA‐C2/C6 Resins in Solid‐Phase Peptide Synthesis

#### 2.4.1 Synthesis of Model Peptide YGGFL

The model pentapeptide Leu‐enkephalin (YGGFL) was synthesized on newly developed Seplife PA C2 and C6 polyacrylate resins using Fmoc‐based SPPS. Coupling was performed by pre‐activating Fmoc‐Aaa‐OH with DIC and OxymaPure (3 eq. each) in 0.2 mL of solvent for 3 min. The mixture was then added to the resin and allowed to react for 1h or longer depend on the synthesis. Fmoc removal was carried out with 0.3 mL of piperidine‐DMF (2:8) (see below for conditions). After coupling and Fmoc removal, the resin was washed with DMF (0.3 mL x 1). Upon completion of the synthesis, the resin bound peptide was washed with MeOH and dried. Cleavage from the resin and side chain deprotection were carried out using TFA‐TIS‐H_2_O (95:2.5:2.5) for 1h, RT. The cleaved peptide was precipitated with cold cyclopentylmethyl ether (CPME), follow by centrifugation, and removal of the supernatant by decantation. The final crude peptide was dissolved in water and analysed by both high‐performance liquid chromatography (HPLC) and liquid chromatography‐mass spectrometry (LC‐MS). Since the resins did not show swelling in common solvents, the first synthesis was carried out under standard conditions to evaluate their performance using DMF as solvent. Two protocols were compared: (i) 1 + 7 min for Fmoc removal with 1h coupling and (ii) 30 min for Fmoc removal with 2 h coupling.

**FIGURE 3:**
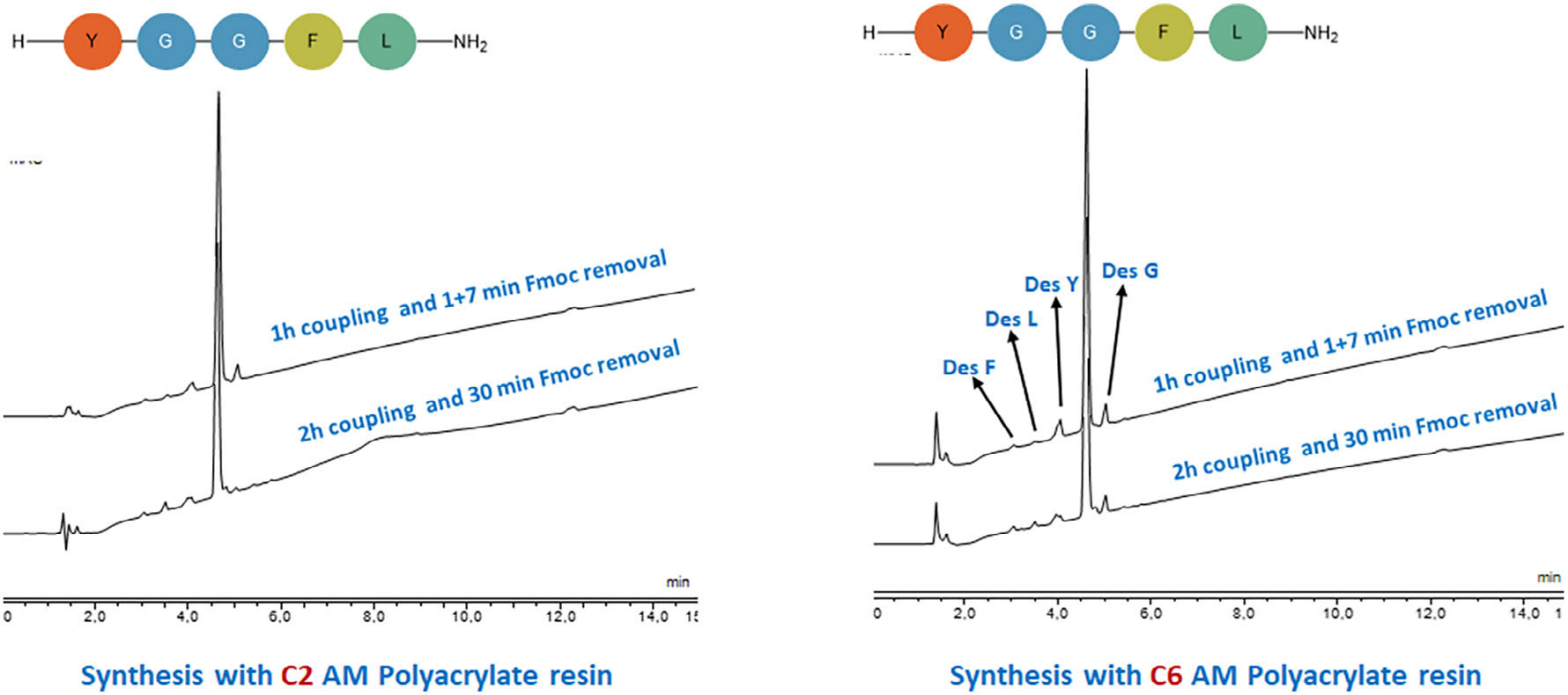
HPLC of YGGFL‐NH_2_ Synthesis on PA‐C6 versus PA‐C2 resin; gradient 5%–95% B into A in 15 min; flow rate: 1 mL/min; detection at 220 nm.

HPLC analysis showed no practical differences between the two protocols (Figure 3). On the PA‐C6 resin, the shorter protocol produced several deletion products, including deletion of Phe (Des F), Leu (Des L), Tyr (Des Y), and Gly (Des G). Extending both the Fmoc removal and coupling time gave a peptide with less impurity. This suggests that the PA‐C6 longer spacer resin requires more demanding conditions to ensure efficient Fmoc removal and complete coupling. On the PA‐C2 resin, the peptide purity was slightly better. Deletion sequences were less compared with the PA‐C6 resin, indicating that the C2 shorter spacer resin improves reagent access and coupling efficiency. Nevertheless, the extended 30 min/2h protocol still provide the best result with approximately 80% purity, and minimal side peaks. While the purity of the final peptide was acceptable, the recovery was only around 50%. This was also found in the rest of model peptides synthesized. This should be further investigated.

#### 2.4.2 Solvent Minimization and the Use of Other Green Solvents for Enabling Sustainable Solid‐Phase Peptide Synthesis

Reducing both the hazardous and volume of solvents used in SPPS can significantly lower the environmental impact of peptide manufacturing. In this study, we evaluated several solvents with improved environmental or safety profiles compared to DMF, TEP, MeCN, EtOAc, acetone, 2‐MeTHF, and THF. Although MeCN and THF are not classified as green solvents, they are notably less hazardous than DMF and were therefore included as intermediate options. Using these solvents, we synthesized a set of model peptides to assess their performance and suitability for more sustainable SPPS workflows. Those include Leu‐enkephalinamide (YGGFL‐NH_2_), the linear decapeptide C‐terminal fragment of tirzepatide (GPSSGAPPPS‐NH_2_), an analogue of afamelanotide (SYSLEHFRWGKPV‐NH_2_), and angiotensin (DRVYIHPF‐NH_2_). Each coupling cycle utilized 3 eq. of Fmoc‐AA‐OH, DIC, and OxymaPure, with pre‐activation in 0.5 mL of the selected solvent for 3 min prior to transfer into the reactor. Coupling was carried out at 45°C for 1h. Post‐coupling, resins were washed three times with 0.5 mL of the same solvent. Fmoc removal was performed using 0.3 mL of 20% piperidine in the respective solvent for 1 + 10 min, followed by three washes with 0.3 mL of the solvent. Crude peptides were cleaved, dissolved in water, and characterized by HPLC and LCMS.

#### 2.4.3 Synthesis of YGGFL‐NH_2_ with Seplife PA‐C2/C6 Resin

**FIGURE 4:**
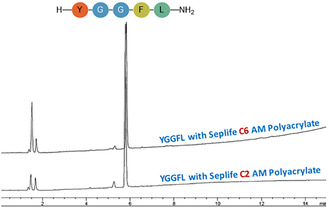
Synthesis of Leu‐Enkephalinamide at 45°C on PA‐C6/PA‐C2 resin. Gradient: 5%–95% B into A in 15 min; flow rate: 1 mL/min; detection at 220 nm.

First, the Leu‐enkephalinamide was synthesized using Seplife PA‐C2 and PA‐C6 resin in DMF as solvent as reference. Figure 4 of the HPLC of crude peptides shows that the use of heating resulted in high purity (97%) for both resins in comparison with RT (Figure 3). The main peak corresponding to the target Leu‐enkephalinamide, with minimal side products, and no deletion sequences were detected. As both resins gave similar results, the study was continued with Seplife PA‐C2 resin (see Figures S16–S18).

**FIGURE 5:**
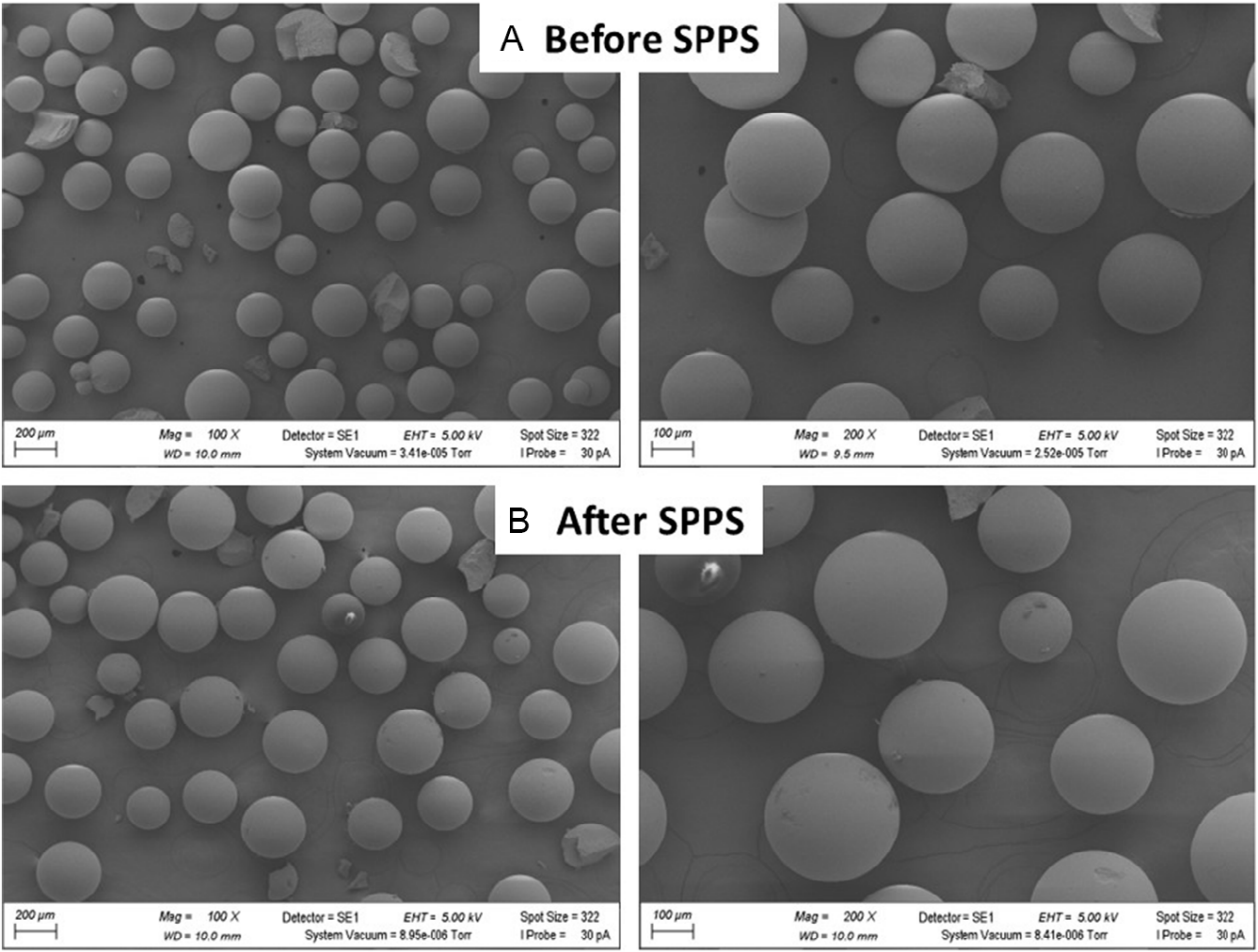
SEM images for Seplife PA‐C2. (A) Before SPPS and (B) after SPPS (of peptidyl resin).

**FIGURE 6:**
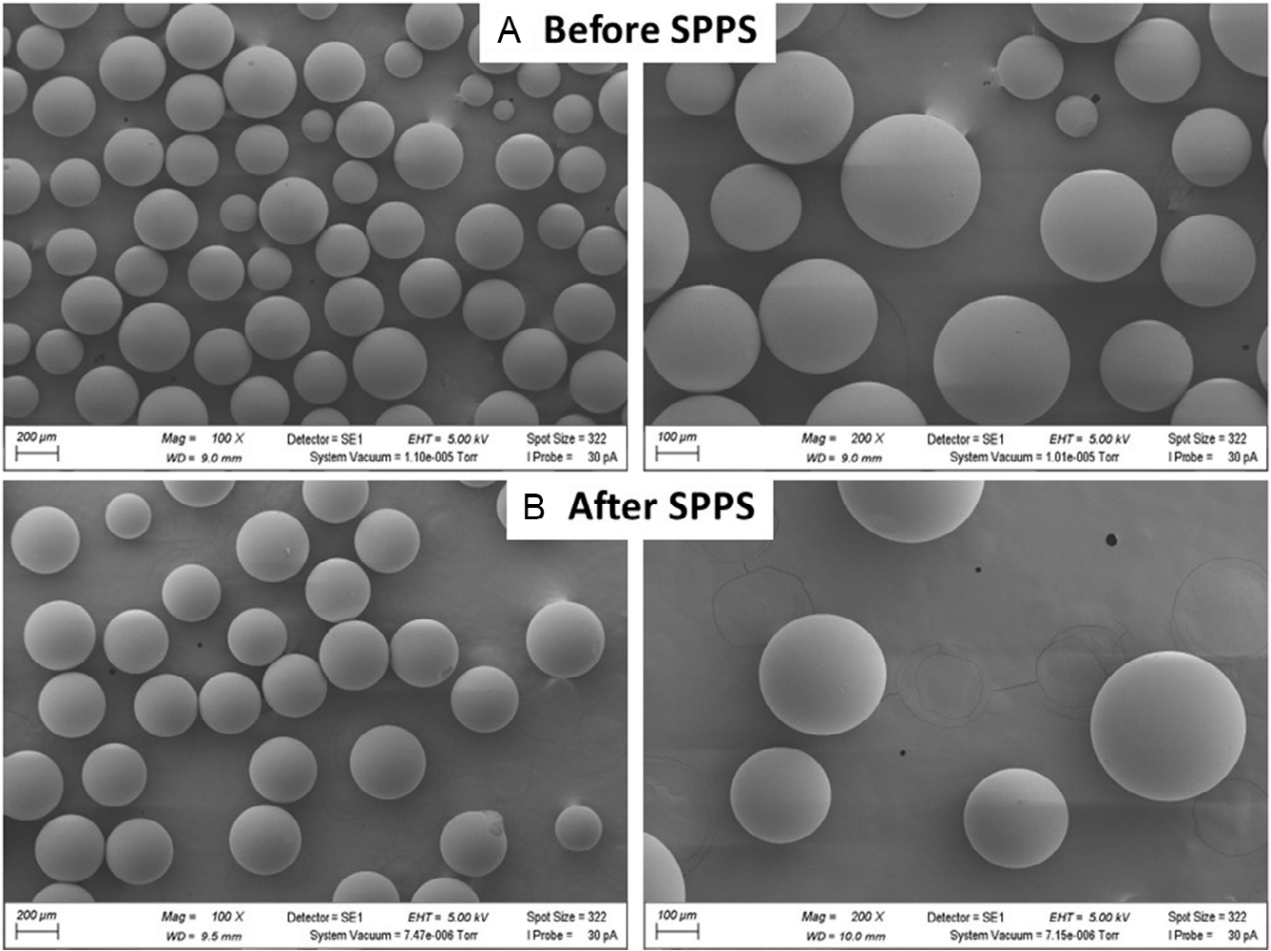
SEM images for Seplife PA‐C6. (A) Before SPPS and (B) after SPPS (of peptidyl resin).

Scanning electron microscopy (SEM) images were acquired to evaluate the morphological integrity of the Seplife resins. Figure 5 presents the resin morphology before and after SPPS for Seplife PA‐C2, while Figure 6 shows the corresponding images for Seplife PA‐C6. In both cases, no noticeable morphological degradation was observed after SPPS, indicating that the resin structure remains intact and is not adversely affected by solvent exposure.

#### 2.4.4 Synthesis of Angiotensin (DRVYIHPF‐NH_2_) on Seplife PA‐C2 Resin

**FIGURE 7:**
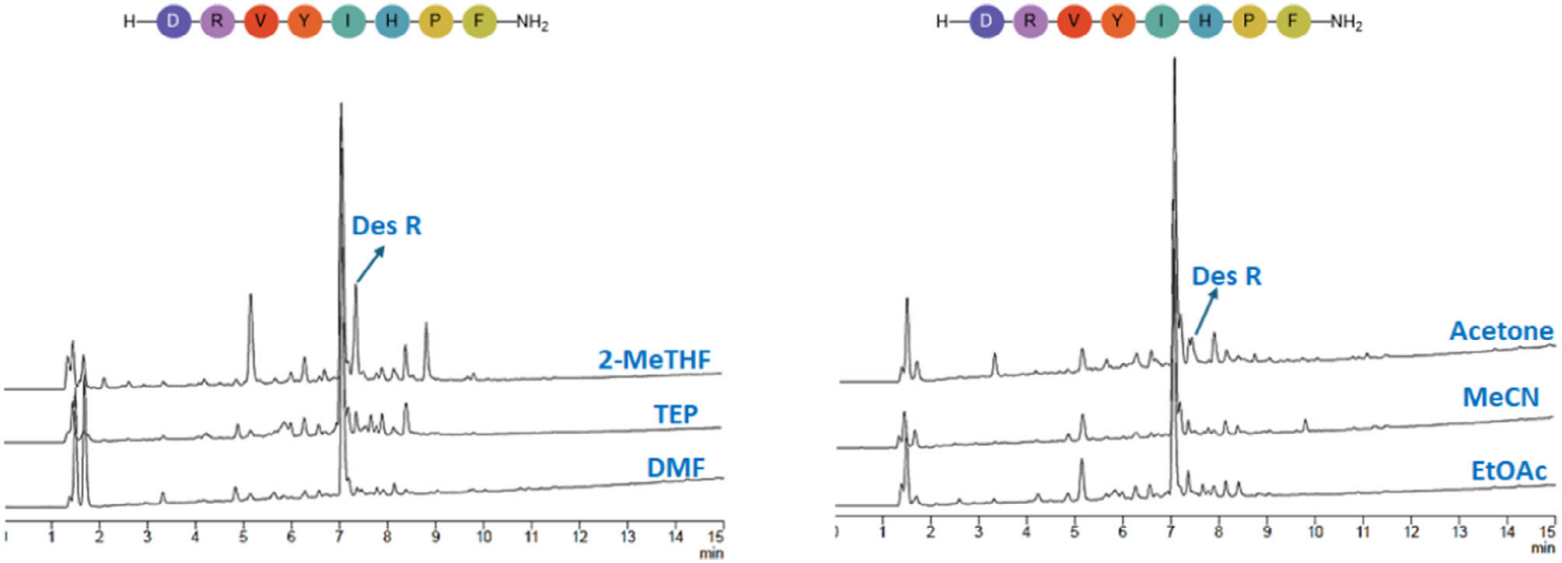
Synthesis of H‐DRVYIHPF‐NH_2_ at 45°C on PA‐C2 resin, gradient: 5%–60% B into A in 15 min; flow rate:1 mL/min; detection at 220 nm.

The peptide angiotensin (H‐DRVYIHPF‐NH_2_) was synthesized on Seplife PA‐C2 resin using the following solvents: DMF, TEP, MeCN, EtOAc, acetone, and 2‐MeTHF. The resulting products were analysed by HPLC (Figure 7). Amongst the tested solvents, MeCN afforded the highest product purity (84.5%), followed by EtOAc (77.7%), TEP (75.3%), and acetone (74.4%), with minimal deletion of the Arg residue (Des R). In contrast, peptide synthesis in 2‐MeTHF (50.2%) resulted in extensive Arg deletion and the appearance of several major side products peaks (see Figure SI‐19 to SI‐22).

#### 2.4.5 Synthesis of an Analogue of Afamelanotide (H‐SYSLEHFRWGKPV‐NH_2_) on PA‐C2 Resin

**FIGURE 8:**
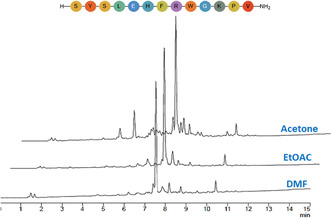
Synthesis of H‐SYSLEHFRWGKPV‐NH_2_ at 45°C on PA‐C2 resin, gradient: 5%–60% B into A in 15 min; flow rate: 1 mL/min; detection at 220 nm.

Using Seplife PA‐C2 resin, the synthesis of an analogue of afamelanotide (H‐SYSLEHFRWGKPV‐NH_2_) was carried out with EtOAc, acetone, and DMF, the latter used for comparison. Analysis of the final crude products by HPLC (Figure 8) indicated that the synthesis carried out with EtOAc (70%) gave similar results when compared with DMF (71.6%). The synthesis in acetone (55.5%) gave substantially less purity, although the main peak was clearly corresponded to the target peptide. This suggests that afamelanotide (SYSLEHFRWGKPV‐NH_2_) can be successfully synthesized in a greener low‐toxicity EtOAc and acetone. (See Figures S23–S24).

#### 2.4.6 Synthesis of GPSSGAPPPS‐NH_2_ on Seplife PA‐C2 Resin

Next, the C‐terminal decapeptide fragment of tirzepatide (H‐GPSSGAPPPS‐NH_2_) was attempted. The synthesis was again carried out in various solvents (DMF, THF, MeCN, and 2‐MeTHF) using for both treatment and washing five volumes of solvents with respect to the mass of resin. As shown in Figure S25, amongst the solvents tested, MeCN produced the highest crude purity (95.8%), giving results similar to those obtained with DMF (97.4%). This was followed by 2‐MeTHF (92.9%) and THF (86.1%). These findings confirm the potential of using the rigid Seplife PA‐C2 resin in combination with greener solvents than DMF (see Figures S26–S8).

**FIGURE 9:**
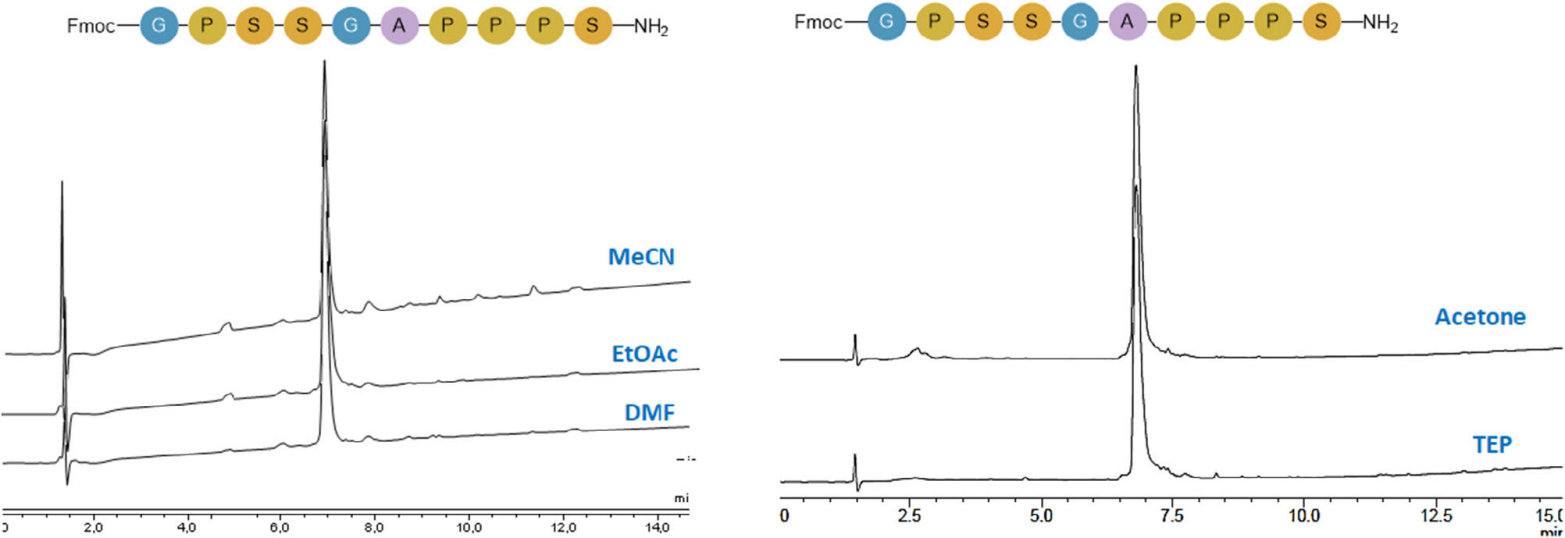
Synthesis of Fmoc‐GPSSGAPPPS‐NH_2_ at 45°C on PA‐C2 resin, gradient: 5%–95% B into A in 15 min; flow rate: 1 mL/min; detection at 220 nm.

Next, the synthesis of the same peptide but with the last Fmoc on (Fmoc‐GPSSGAPPPS‐NH_2_), introduced to provide slightly more hydrophobicity to the peptide and facilitate its HPLC analysis, was synthesized using DMF, EtOAc, MeCN, TEP, and acetone as solvents. In all four cases, the quality of the target peptide was excellent (90%–95%) (Figure 9). Acetone gave, in this case, the best purity (95.8%), a result likely linked to its moderate polarity and low viscosity, which promote faster diffusion of activated species through the rigid, non‐swelling polyacrylate resin matrix. It is also important to highlight the purity obtained when the synthesis was carried out with TEP (94%)%) (see Figures S29–S31). This outcome is particularly noteworthy considering the high polarity and higher viscosity of TEP, which can slow reagent mobility; nevertheless, its strong solvating capability for coupling reagents appears to offset these limitations when used with the Seplife PA‐C2 resin. This is even more impressive when compared with the performance of TEP on PS‐DVB resin, where only a pentapeptide (Leu‐enkephalinamide analogue) could be synthesized [27].

Process mass intensity (PMI) has also been calculated to depict the sustainability of the process. Table 2 shows the PMI calculated in each of the above cases using the tool developed by our research group and will be published elsewhere.

**TABLE 2:** PMI calculated for the synthesis of peptides using Seplife PA‐C2 and Seplife PA‐C6 resins.

Sequence	PMI	Sequence	PMI
YGGFL‐C2‐in DMF	1892	SYSLEHFRWGKPV‐C2‐DMF	1073
YGGFL‐C6‐in DMF	1338	SYSLEHFRWGKPV‐C2‐EtOAc	1099
DRVYIHPF‐C2‐in DMF	1000	SYSLEHFRWGKPV‐C2‐acetone	1063
DRVYIHPF‐C2‐in TEP	1312	GPSSGAPPPS‐C2‐DMF	1505
DRVYIHPF‐C2‐in MeCN	945	GPSSGAPPPS‐C2‐MeCN	1437
DRVYIHPF‐C2‐in EtOAc	1247	GPSSGAPPPS‐C2‐THF	1916
DRVYIHPF‐C2‐in acetone	1162	GPSSGAPPPS‐C2‐2Me‐THF	1403
DRVYIHPF‐C2‐in 2‐MeTHF	1666		

## 3 Conclusion

The most important conclusion of this work is that the challenge of greening SPPS must be approached from a multifactorial perspective. Until now, our group and others have attempted to identify a single solvent capable of replacing DMF while relying exclusively on PS‐DVB resins a task that has proven arduous and ultimately limiting. We propose that the most effective path towards greener SPPS is to identify resin–solvent combinations that can simultaneously support the two key reactions involved in peptide synthesis: coupling and Fmoc removal.

In this context, we advocate the use of relatively rigid, non‐swelling resins. With such resins, swelling is no longer a determining factor, allowing the use of solvents that do not swell the solid support and thereby greatly expanding the range of green solvents that can be evaluated. Traditionally, the first requirement for any potential green solvent or solvent mixture has been its ability to swell PS‐DVB. The adoption of non‐swelling resins removes this fundamental restriction.

Following this principle, we have demonstrated that peptides can be synthesized using acetone or EtOAc as single solvents. To the best of our knowledge, this is the first time these solvents have been used independently for peptide synthesis. Although not entirely green, MeCN also showed good performance, consistent with earlier results from our group demonstrating that MeCN can be an effective solvent for SPPS when PEG‐based resins known to swell better in MeCN than conventional PS‐DVB are employed [28]. In other cases, TEP and 2‐MeTHF also afforded excellent results. One limitation of the current system is the relatively low peptide recovery after global deprotection and cleavage, which is approximately 50%. This issue warrants further investigation.

An additional advantage of using non‐swelling resins is the reduction of solvent consumption by at least 50%, as no extra solvent is required to accommodate resin swelling. A further benefit is that the volume of the peptide resin remains constant throughout the synthesis, simplifying handling and process control.

Overall, the results presented here open new avenues for increasing the sustainability of SPPS, reaffirming the recent findings of Biondi and co‐workers [24]. The process of greening SPPS must be approached from a multifactorial perspective, using different resins, solvents, and reagents than those used in conventional SPPS chemistry. Work is currently underway in our laboratories to explore new resins, greener solvents, and resin–solvent pairings that may further improve the environmental footprint of peptide manufacturing

## 4 Experimental Part

### 4.1 Materials and Method

DMSO, MeCN, EtOAc, acetone, DMF, THF, and 2‐MeTHF were purchased from Honeywell, while OxymaPure, *N*, *N’*‐diisopropylcarbodiimide (DIC) and TEP were a generous gift from Luxembourg Biotech (Rehovot, Israel). All chemicals and solvents were acquired from commercial sources and used without further purification. Fmoc‐amino acids [Fmoc‐Aaa(PG)‐OH] were sourced from Iris Biotech. Amino‐functionalized polyacrylate resins bearing either a short C2 or a longer C6 aliphatic spacer were used as solid supports, providing primary amine groups for anchoring the Rink linker prior to peptide assembly. Seplife PA‐C2/C6 were manufactured using a priority method by Sunresin (Xi’an, China). Piperidine was purchased from Sigma‐Aldrich. Additional organic solvents and HPLC‐grade acetonitrile (MeCN) were obtained from Merck. Milli‐Q water was used for reverse‐phase high‐performance liquid chromatography (RP‐HPLC) analyses. Analytical HPLC was performed on Shimadzu‐LC‐2050 system equipped with a Phenomenex AerisC18 (3.6 μm, 4.6 × 150 mm) column, operating at a flow rate of 1.0 mL/min with UV detection at 220 nm. Data were processed using LabSolutions software. Buffer A consisted of 0.1% trifluoroacetic acid (TFA) in water, and Buffer B was 0.1% TFA in MeCN. LC‐MS was carried out on a Thermo Scientific Dionex UltiMate 3000 with a Phenomenex AerisC18 (3.6 μm, 4.6 × 150 mm) column. Buffer A was 0.1% formic acid in water, and Buffer B was 0.1% formic acid in MeCN.

### 4.2 Solid‐Phase Peptide Synthesis

All peptides were synthesized using a standard Fmoc/*t*Bu based SPPS methodology. Synthesis was performed in a polypropylene syringe reactor fitted with a polyethylene filter.

#### 4.2.1 Resin Preparation

Seplife Fmoc‐RinkAmide‐PA‐C2/C6 (loading: 0.4 mmol/g; 0.05‐mmol scale) were used as the solid support for the peptide assembly. The resin was washed with respective solvent (0.2 mL x 2).

#### 4.2.2 Fmoc Removal

The Fmoc protecting group was removal using 0.3 mL 20% piperidine in the chosen solvent (1 x 1 min, 1 x 7 min) unless otherwise specified. The reaction mixture was drained, and the resin was washed with selected solvent (0.3 mL x 2).

#### 4.2.3 Fmoc‐Aaa‐OH Coupling

For each coupling step, Fmoc‐Aaa‐OH (3 eq.), OxymaPure (3 eq.), and DIC (3 eq.) were dissolved in the chosen green solvent (0.2 mL). After a pre‐activation period of 3–5 min, the mixture was added to the resin, agitated on shaker for 1 h at RT/45°C, unless otherwise stated. Following coupling, the mixture was drained, and the resin was washed with solvent (0.3 mL x 1). Fmoc was then removed as explained above. This process was repeated sequentially until the complete peptide chain was assembled.

#### 4.2.4 Cleavage and Global Deprotection

Upon completion of chain assembly, the peptide resin was washed with methanol (0.3 mL x 2) and dried. The obtained dried peptidyl resin was treated with 1 mL cleavage cocktail composed of TFA‐TIS‐H_2_O in a ratio of 95:2.5:2.5 (*v/v/v*) for 1 h at RT. The crude peptide was precipitated by the addition of cold CPME (10 mL x 3) to the cleavage mixture. The suspension was centrifuged, and the supernatant was discarded. The resulting peptide resin pellet was extracted with water and filtered to remove residual resin, and the aqueous phase was lyophilized to yield the crude peptide.

## Supporting Information

Additional supporting information can be found online in the Supporting Information section. **Supporting Fig. S1**: Solubility study of Fmoc‐Leu‐OH and Fmoc‐Phe‐OH at 0.6 M. **Supporting Fig. S2**: Solubility study of Fmoc‐Gly‐OH and Fmoc‐Tyr(tBu)‐OH at 0.6 M. **Supporting Fig. S3**: Solubility study of Fmoc‐Ser(tBu)‐OH and Fmoc‐Pro‐OH at 0.6 M. **Supporting Fig. S4**: Solubility study of Fmoc‐Ala‐OH and Fmoc‐Val‐OH at 0.6 M. **Supporting Fig. S5**: Solubility study of Fmoc‐Lys(Boc)‐OH and Fmoc‐Trp(Boc)‐OH at 0.6 M. **Supporting Fig. S6**: Solubility study of Fmoc‐Arg(Pbf)‐OH and Fmoc‐His(Trt)‐OH at 0.6 M. **Supporting Fig. S7**: Solubility study of Fmoc‐Glu(O*t*Bu)‐OH and Fmoc‐Ile‐OH at 0.6 M. **Supporting Fig. S8**: Solubility study of Fmoc‐Asp(O*t*Bu)‐OH at 0.6 M. **Supporting Fig. S9**: Solubility study of Fmoc‐Leu‐OH and Fmoc‐Phe‐OH at 0.2 M. **Supporting Fig. S10**: Solubility study of Fmoc‐Gly‐OH and Fmoc‐Tyr(tBu)‐OH at 0.2 M. **Supporting Fig. S11**: Solubility study of Fmoc‐Ser(tBu)‐OH and Fmoc‐Pro‐OH at 0.2 M. **Supporting Fig. S12**: Solubility study of Fmoc‐Ala‐OH and Fmoc‐Val‐OH at 0.2 M. **Supporting Fig. S13**: Solubility study of Fmoc‐Lys (Boc)‐OH and Fmoc‐Trp(Boc)‐OH at 0.2 M. **Supporting Fig. S14**: Solubility study of Fmoc‐Arg(Pbf)‐OH and Fmoc‐His(Trt)‐OH at 0.2 M. **Supporting Fig. S15**: Solubility study of Fmoc‐Glu(O*t*Bu)‐OH and Fmoc‐Ile‐OH at 0.2 M. **Supporting Fig. S16**: Solubility study of Fmoc‐Asp(O*t*Bu)‐OH at 0.2 M. **Supporting Fig. S17**: HPLC for Leu‐Enkephalinamide at 45°C. gradient: 5–95 % B into A in 15 min; flow rate: 1 mL/min; detection at 220 nm. **Supporting Fig. S18**: LC‐MS for Leu‐Enkephalinamide at 45°C. **Supporting Fig. S19**: HPLC for angiotensin at 45°C in MeCN and EtOAc. **Supporting Fig. S20**: HPLC for angiotensin at 45°C in acetone and TEP. **Supporting Fig. S21**: HPLC for angiotensin at 45°C in 2‐MeTHF and DMF. **Supporting Fig. S22**: LC‐MS for angiotensin (DRVYIHPF) and Des R (DVYIHPF). **Supporting Fig. S23**: HPLC for Afamelanotide SYSLEHFRWGKPV‐NH_2_ in DMF and EtOAc. **Supporting Fig. S24**: HPLC for Afamelanotide SYSLEHFRWGKPV‐NH_2_ in acetone. **Supporting Fig. S25**: Synthesis of H‐GPSSGAPPPS‐NH_2_ at 45°C. gradient: 0–30 % B into A in 15 min; flow rate: 1 mL/min; detection at 220 nm. **Supporting Fig. S26**: HPLC for H‐GPSSGAPPPS‐NH_2_ in 2‐MeTHF and MeCN. **Supporting Fig. S27**: HPLC for H‐GPSSGAPPPS‐NH_2_ in DMF and THF. **Supporting Fig. S28**: LC‐MS for H‐GPSSGAPPPS‐NH_2_. **Supporting Fig. S29**: HPLC for Fmoc‐GPSSGAPPPS‐NH_2_ in acetone and TEP. **Supporting Fig. S30**: HPLC for Fmoc‐GPSSGAPPPS‐NH_2_ in MeCN, EtOAc and DMF. **Supporting Fig. S31**: LC‐MS for Fmoc‐GPSSGAPPPS‐NH_2_.

## Conflicts of Interest

Co‐authors working in Sunresin declare that Sunresin could commercialize these resins. The rest of authors declare non‐conflict of interest.

## Supporting information

Supplementary Material

## Data Availability

The data that support the findings of this study are available on request from the corresponding author. The data are not publicly available due to privacy or ethical restrictions.

## References

[1] V. du Vigneaud , C. Ressler , J. M. Swan , C. W. Roberts , and P. G. Katsoyannis , “The Synthesis of Oxytocin ^1^ ,” Journal of the American Chemical Society 76, no. 12 (1954): 3115–3121.

[2] T. Bruckdorfer , O. Marder , and F. Albericio , “From Production of Peptides in Milligram Amounts for Research to Multi‐Tons Quantities for Drugs of the Future,” Current Pharmaceutical Biotechnology 5, no. 1 (2004): 29–43. 14965208 10.2174/1389201043489620

[3] A. A. Zompra , A. S. Galanis , O. Werbitzky , and F. Albericio , “Manufacturing Peptides as Active Pharmaceutical Ingredients,” Future Medicinal Chemistry 1, no. 2 (2009): 361–377. 21425973 10.4155/fmc.09.23

[4] M. Muttenthaler , G. F. King , D. J. Adams , and P. F. Alewood , “Trends in Peptide Drug Discovery,” Nature Reviews Drug Discovery 20, no. 4 (2021): 309–325. 10.1038/s41573-020-00135-8 33536635

[5] R. B. Merrifield , “Solid Phase Peptide Synthesis. I. The Synthesis of a Tetrapeptide,” Journal of the American Chemical Society 85, no. 14 (1963): 2149–2154.

[6] G. R. Marshall , “The Early Years—Across the Bench from Bruce (1963‐1966,” Peptide Science 90, no. 3 (2008): 190–199. 17941005 10.1002/bip.20870

[7] B. L. Bray , “Large‐Scale Manufacture of Peptide Therapeutics by Chemical Synthesis,” Nature Reviews Drug Discovery 2, no. 7 (2003): 587–593. 12815383 10.1038/nrd1133

[8] M. O. Frederick , R. A. Boyse , T. M. Braden , et al., “Kilogram‐Scale GMP Manufacture of Tirzepatide Using a Hybrid SPPS/LPPS Approach with Continuous Manufacturing,”Organic Process Research & Development 25, no. 7 (2021): 1628–1636.

[9] A. Sharma , A. Kumar , B. G. de la Torre , and F. Albericio , “Liquid‐Phase Peptide Synthesis (LPPS): A Third Wave for the Preparation of Peptides,” Chemical Reviews 122, no. 16 (2022): 13516–13546. 35816287 10.1021/acs.chemrev.2c00132

[10] J. M. Collins , S. K. Singh , T. A. White , et al., “Total Wash Elimination for Solid Phase Peptide Synthesis,” Nature Communications 14, no. 1 (2023): 8168. 10.1038/s41467-023-44074-5 PMC10710472 38071224

[11] A. Kumar , A. Sharma , B. G. de la Torre , and F. Albericio , “In Situ Fmoc Removal – a Sustainable Solid‐Phase Peptide Synthesis Approach,” Green Chemistry 24, no. 12 (2022): 4887–4896.

[12] F. Garcia‐Martin and F. Albericio , “Solid Supports for the Synthesis of Peptides. From First Used Resin to the Most Sophisticated in the Market,” Chemistry Today 26, no. 4 (2008): 29–34.

[13] S. Ramkisson , Y. E. Jad , A. Sharma , B. G. de la Torre , and F. Albericio , “Octagel Resin ‐ a New PEG‐PS‐Based Solid Support for Solid‐Phase Peptide Synthesis,” Letters in Organic Chemistry 16, no. 12 (2019): 935–940.

[14] M. Renil , M. Ferreras , J. M. Delaisse , N. T. Foged , and M. Meldal , “PEGA Supports for Combinatorial Peptide Synthesis and Solid‐Phase Enzymatic Library Assays,” Journal of Peptide Science 4, no. 3 (1998): 195–210. 9643628 10.1002/(SICI)1099-1387(199805)4:3%3C195::AID-PSC141%3E3.0.CO;2-R

[15] F. Garcia‐Martin , M. Quintanar‐Audelo , Y. Garcia‐Ramos , et al., “ChemMatrix, a Poly(ethylene Glycol)‐Based Support for the Solid‐Phase Synthesis of Complex Peptides,” Journal of Combinatorial Chemistry 8, no. 2 (2006): 213–220. 16529516 10.1021/cc0600019

[16] M. L. Ramsing , C. Warming , and M. Meldal , “Green Resins for All: Sustainable Preparation of PEGA Resin for Peptide and Protein Synthesis and Immobilization,” ACS Applied Materials & Interfaces 17, no. 17 (2025): 25764–25773. 40248980 10.1021/acsami.5c01951

[17] B. G. de la Torre , A. Jakab , and D. Andreu , “Polyethyleneglycol‐Based Resins as Solid Supports for the Synthesis of Difficult or Long Peptides,” International Journal of Peptide Research and Therapeutics 13, no. 1‐2 (2007): 265–270.

[18] S. Sano , R. Tokunaga , and K. A. Kun , “Solid‐Phase Method for Peptide Synthesis Using Macroreticular Copolymers,” Biochimica et Biophysica Acta (BBA) ‐ General Subjects 244, no. 1 (1971): 201–205. 5120215 10.1016/0304-4165(71)90137-1

[19] A. Losse , “Über Den Einsatz Makroporöser Harze in der Festphasen‐Peptidsynthese,” Tetrahedron 29, no. 9 (1973): 1203–1208.

[20] F. Albericio , M. Pons , E. Pedroso , and E. Giralt , “Comparative Study of Supports for Solid‐Phase Coupling of Protected‐Peptide Segments,” The Journal of Organic Chemistry 54, no. 2 (1989): 360–366.

[21] W. Parr and K. Grohmann , “A New Solid Support for Polypeptide Synthesis,” Tetrahedron Letters 12, no. 28 (1971): 2633–2636.

[22] R. A. Houghten and Y. Yu , “Volatilizable” Supports for High‐Throughput Organic Synthesis,” Journal of the American Chemical Society 127, no. 24 (2005): 8582–8583. 15954749 10.1021/ja0402396

[23] L. Sinigoi , P. Bravin , C. Ebert , et al., “Synbeads Porous‐Rigid Methacrylic Support: Application to Solid Phase Peptide Synthesis and Characterization of the Polymeric Matrix by FTIR Microspectroscopy and High Resolution Magic Angle Spinning NMR,” Journal of Combinatorial Chemistry 11, no. 5 (2009): 835–845. 19594112 10.1021/cc900050t

[24] L. Lastella , M. Zecca , P. Centomo , et al., “Toward a Green SPPS: The Use of an Innovative Mesoporous pDVB Support for Environmentally Friendly Solvents,” Journal of Peptide Science 31, no. 8 (2025): e70038. 40589139 10.1002/psc.70038 PMC12209690

[25] N. Nkwanyana , A. Kumar , A. Chakraborty , et al., “Silica‐Assisted Solid‐Phase Peptide Synthesis (SiPPS,” Journal of Peptide Science 31, no. 9 (2025): e70052. 40817749 10.1002/psc.70052 PMC12357219

[26] O. Al Musaimi , A. Basso , B. G. de la Torre , and F. Albericio , “Calculating Resin Functionalization in Solid‐Phase Peptide Synthesis Using a Standardized Method Based on Fmoc Determination,” ACS Combinatorial Science 21, no. 11 (2019): 717–721. 31610120 10.1021/acscombsci.9b00154

[27] K. P. Nandhini , N. Cele , B. G. de la Torre , and F. Albericio , “Triethyl Phosphate (TEP) as a Green Solvent for Solid‐Phase Peptide Synthesis (SPPS,” Green Chemistry Letters and Reviews 17, no. 1 (2024): 2330639.

[28] G. A. Acosta , M. del Fresno , M. Paradis‐Bas , et al., “Solid‐Phase Peptide Synthesis Using Acetonitrile as a Solvent in Combination with PEG‐Based Resins,” Journal of Peptide Science 15, no. 10 (2009): 629–633. 19634177 10.1002/psc.1158

