# Supplementary material for: Multifactorial Perspective for Greening Solid‐Phase Peptide Synthesis: Rigid Polyacrylate Macroporous Resin in Combination with Green Solvents
Source: ChemSusChem. 2026 Apr 14;19(7):e70591. doi: 10.1002/cssc.70591 (PMC13080234; doi:10.1002/cssc.70591)
Supplement: Supplementary file 1 — Supplementary Material [file CSSC-19-e70591-s001.pdf]

Supporting information

Multifactorial Perspective for Greening Solid-Phase Peptide Synthesis. Rigid Polyacrylate Macroporous Resin in Combination with Green Solvents

Sikabwe Noki[1,2], Ashish Kumar,[1] Cheng Zhang[3], Alesandra Basso[3], Simona Serban[3], Xiaokang Kou[3], Yanjun Li[3], Anamika Sharma,[1] Beatriz G de la Torre[1,2, *], Fernando Albericio[1,4, *]

[1]Peptide Science Laboratory, School of Chemistry and Physics, University of KwaZulu-Natal, Durban 4001, South Africa

[2]KwaZulu-Natal Research Innovation and Sequencing Platform (KRISP), School of Laboratory Medicine and Medical Sciences, College of Health Sciences, University of KwaZulu-Natal, Durban 4041, South Africa

[3] Sunresin New Materials, Sunresin Park, No. 135 Jinye Rd, Xi’an Hi-tech Industrial Development Zone, Shaanxi, 710076, China

[4]CIBER-BBN, Networking Centre on Bioengineering, Biomaterials and Nanomedicine, and Department of Organic Chemistry, University of Barcelona, Martí i Franqués 1-11, 08028-Barcelona, Spain

Corresponding Authors:

Professor Beatriz G de la Torre, KwaZulu-Natal Research Innovation and Sequencing Platform (KRISP), School of Laboratory Medicine and Medical Sciences, College of Health Sciences, UniversityofKwaZulu-Natal, Durban 4041, South Africa. Email: garciadelatorreb@ukzn.ac.za. Phone: +27 614 047 528

Professor Fernando Albericio, Peptide Science Laboratory, School of Chemistry and Physics, University of KwaZulu-Natal, Durban 4001, South Africa. Email: albericio@ukzn.ac.za. Phone: +27 614 009 144

Keywords: Green solvent, polyacrylate macroporous resin, SPPS,

Running Title:

Solubility of Fmoc-aa (PG)-OH in selected solvents at 0.6 M concentration

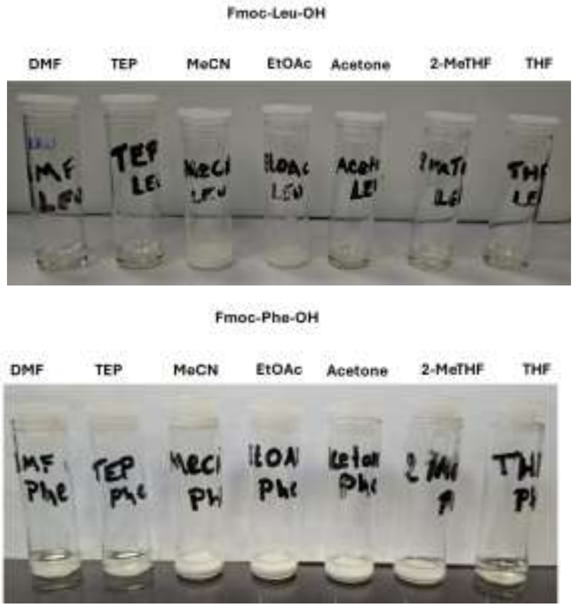

Figure SI-1: Solubility study of Fmoc-Leu-OH and Fmoc-Phe-OH at 0.6 M.

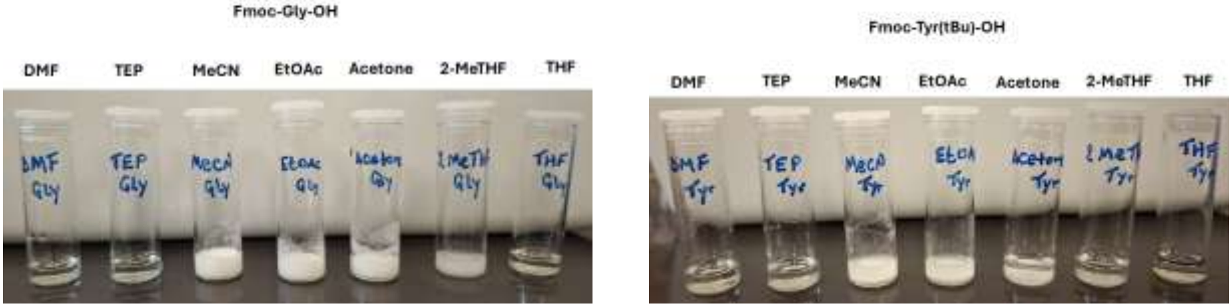

Figure SI-2: Solubility study of Fmoc-Gly-OH and Fmoc-Tyr(tBu)-OH at 0.6 M.

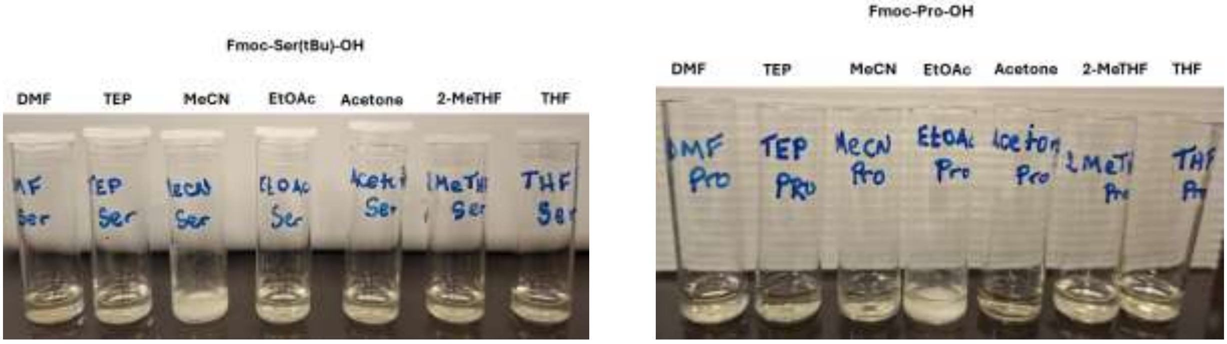

Figure SI-3: Solubility study of Fmoc-Ser(tBu)-OH and Fmoc-Pro-OH at 0.6 M.

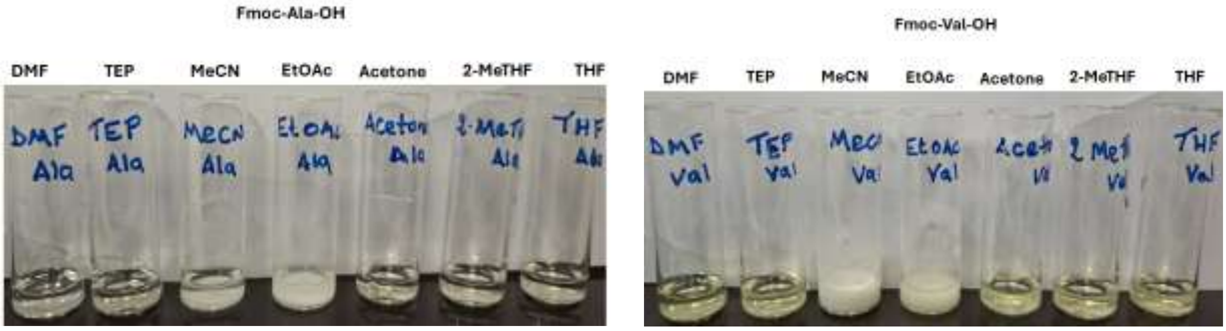

Figure SI-4: Solubility study of Fmoc-Ala-OH and Fmoc-Val-OH at 0.6 M.

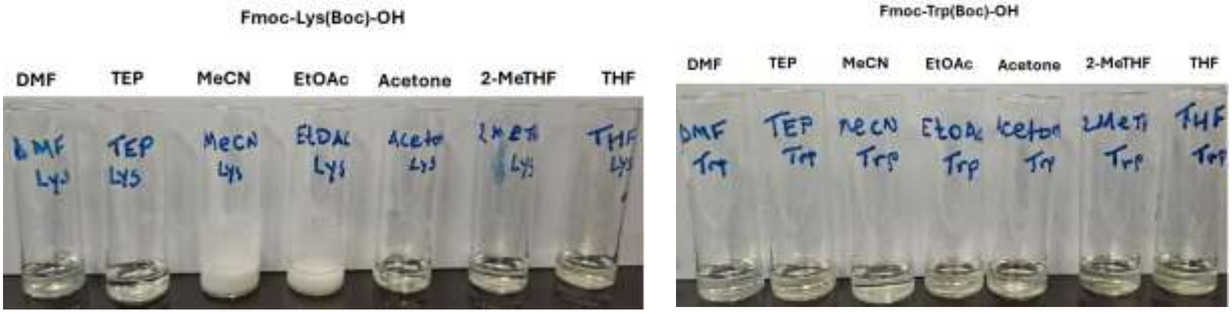

Figure SI-5: Solubility study of Fmoc-Lys(Boc)-OH and Fmoc-Trp(Boc)-OH at 0.6 M.

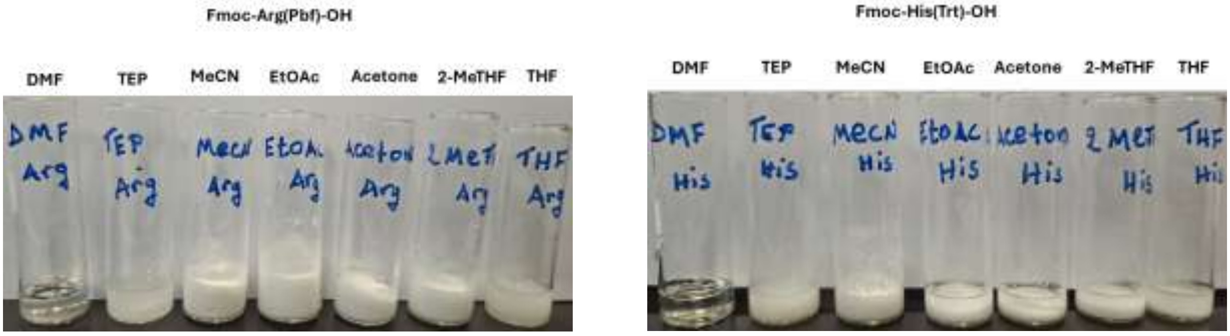

Figure SI-6: Solubility study of Fmoc-Arg(Pbf)-OH and Fmoc-His(Trt)-OH at 0.6 M.

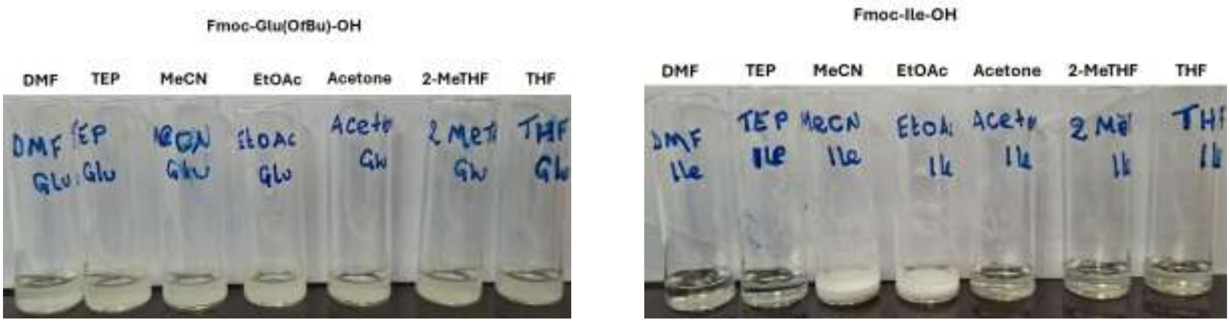

Figure SI-7: Solubility study of Fmoc-Glu(O*t*Bu)-OH and Fmoc-Ile-OH at 0.6 M.

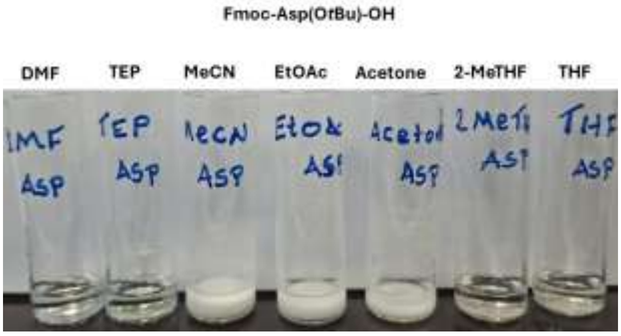

Figure SI-8: Solubility study of Fmoc-Asp(O*t*Bu)-OH at 0.6 M.

Solubility of Fmoc-aa (PG)-OH in selected solvents at 0.2M concentration

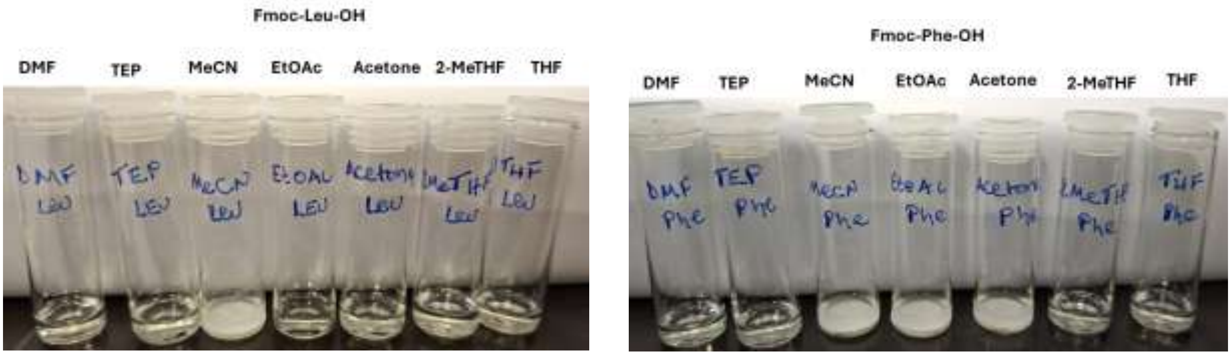

Figure SI-9: Solubility study of Fmoc-Leu-OH and Fmoc-Phe-OH at 0.2 M.

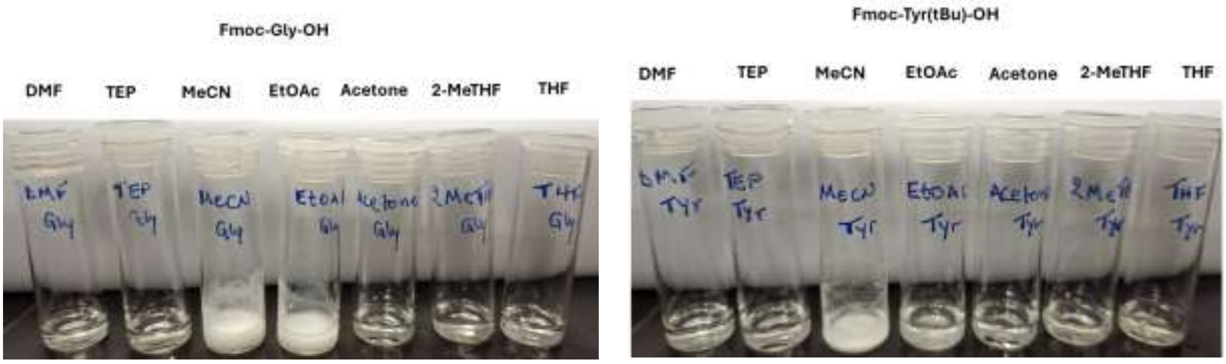

Figure SI-10: Solubility study of Fmoc-Gly-OH and Fmoc-Tyr(tBu)-OH at 0.2 M.

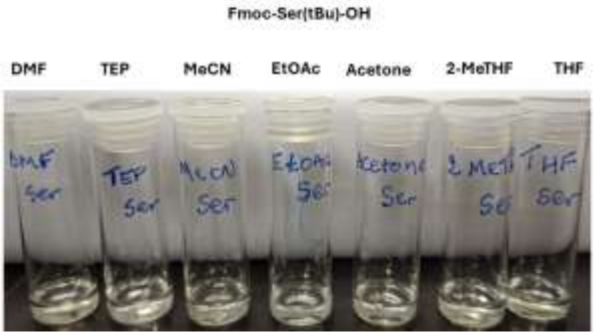

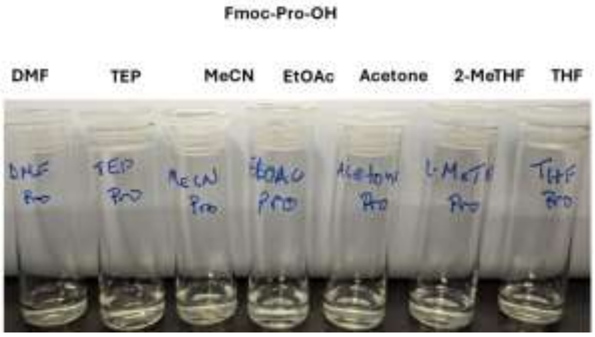

Figure SI-11: Solubility study of Fmoc-Ser(tBu)-OH and Fmoc-Pro-OH at 0.2 M.

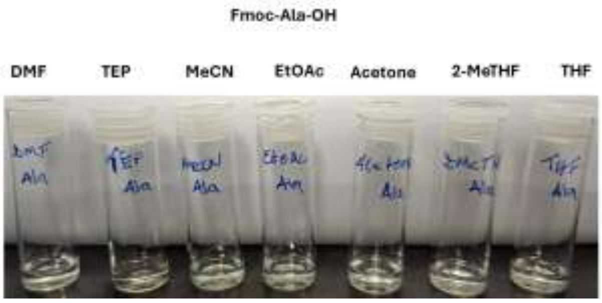

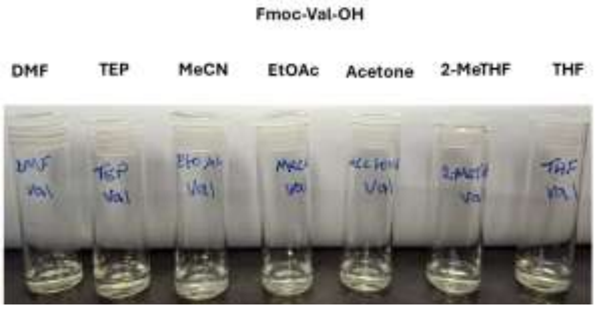

Figure SI-12: Solubility study of Fmoc-Ala-OH and Fmoc-Val-OH at 0.2 M.

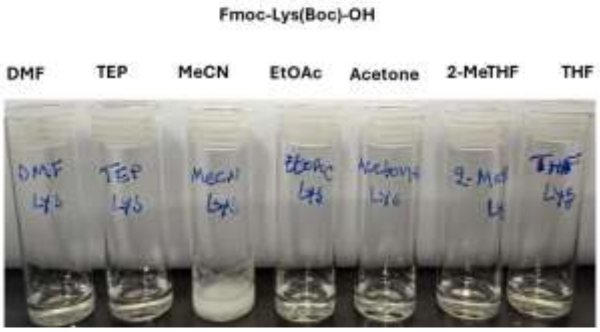

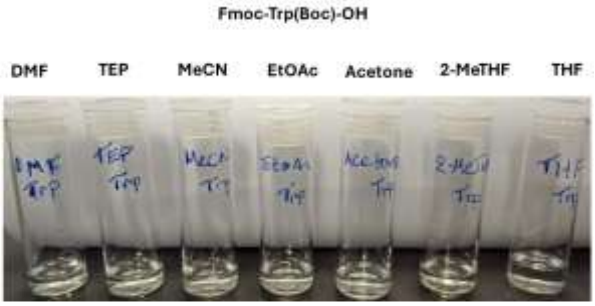

Figure SI-13: Solubility study of Fmoc-Lys (Boc)-OH and Fmoc-Trp(Boc)-OH at 0.2 M.

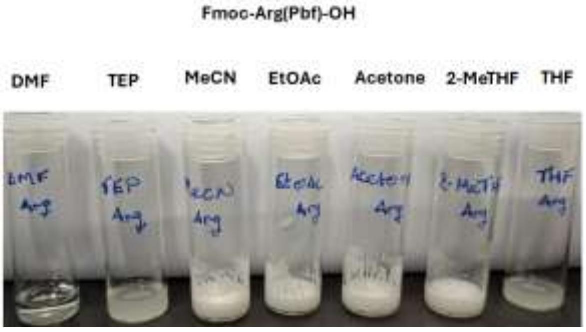

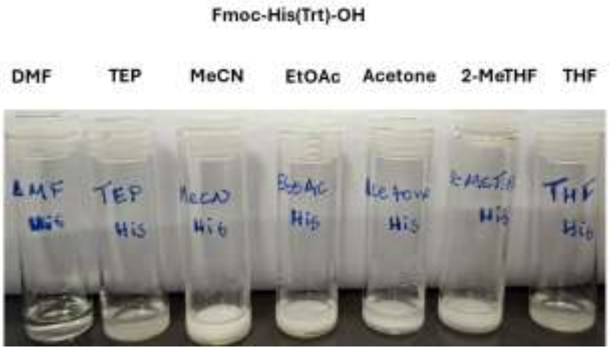

Figure SI-14 Solubility study of Fmoc-Arg(Pbf)-OH and Fmoc-His(Trt)-OH at 0.2 M.

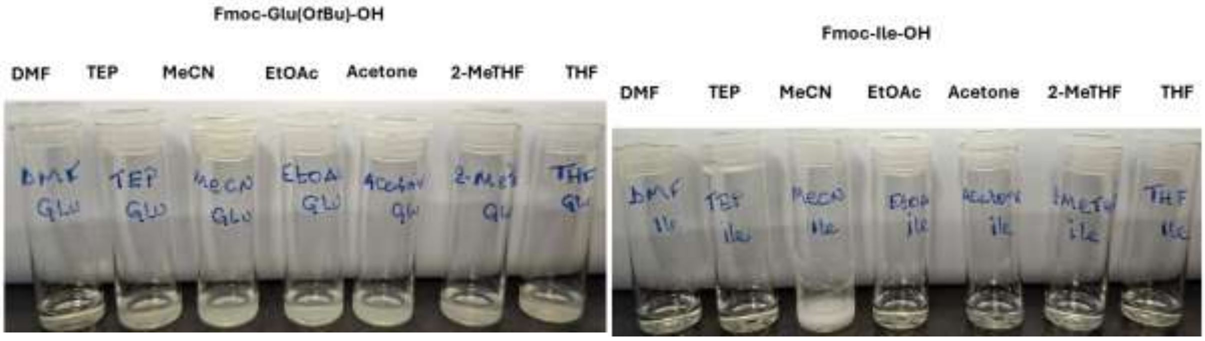

Figure SI-15: Solubility study of Fmoc-Glu(O*t*Bu)-OH and Fmoc-Ile-OH at 0.2 M.

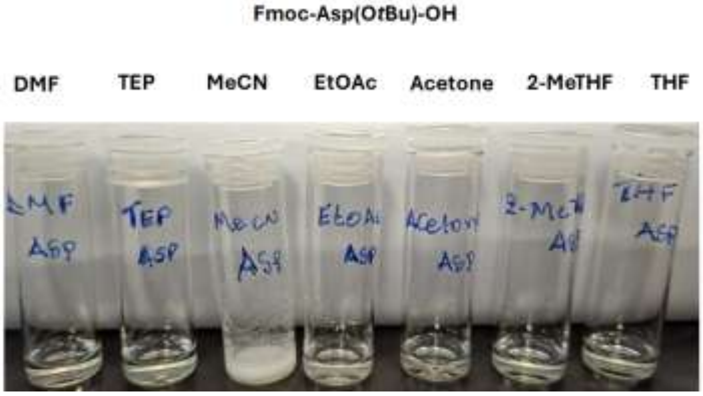

Figure SI-16: Solubility study of Fmoc-Asp(O*t*Bu)-OH at 0.2 M.

Synthesis of YGGFL-NH_2 with C2 and C6 AM polyacrylate resin

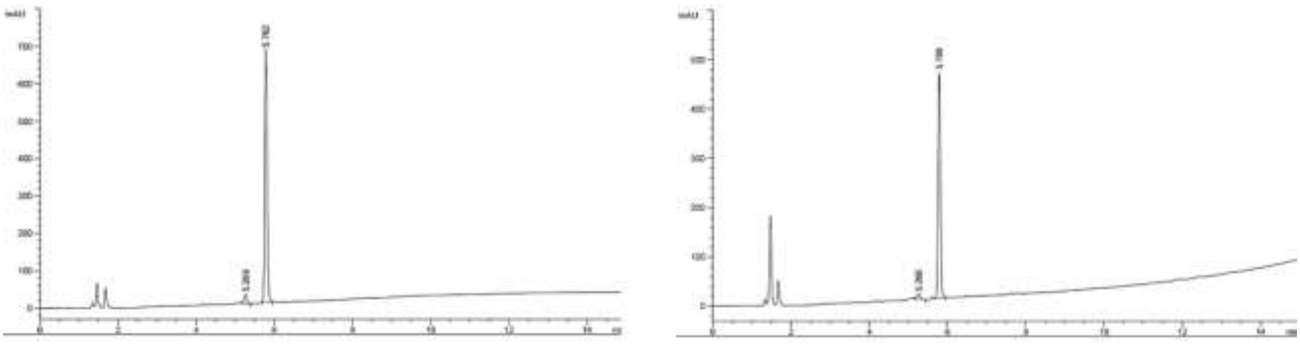

Signal 1: VWD1 A, Wavelength=220 nm, TT

Peak #	RetTime [min]	Type	Width [min]	Area mAU *s	Height [mAU]	Area %
1	5.269	VB	0.0805	133.10956	25.83418	4.3494
2	5.782	BB	0.0680	2927.31958	676.07709	95.6506

YGGFL-NH_2 with Seplife C2 AM Polyacrylate

Signal 1: VWD1 A, Wavelength=220 nm, TT

Peak #	RetTime [min]	Type	Width [min]	Area mAU *s	Height [mAU]	Area %
1	5.266	VB	0.0857	64.71053	11.82067	3.4711
2	5.788	BB	0.0604	1799.52869	458.22562	96.5289

YGGFL-NH_2 with Seplife C6 AM Polyacrylate

Figure SI-17: HPLC for Leu-Enkephalinamide at 45°C. gradient: 5–95 % B into A in 15 min; flow rate: 1 mL/min; detection at 220 nm.

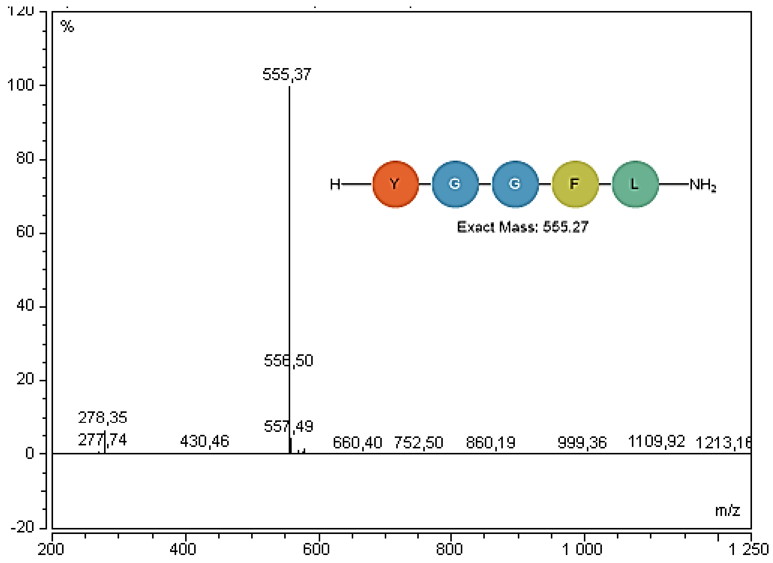

Figure SI-18: LCMS for Leu-Enkephalinamide at 45°C.

Synthesis of angiotensin (DRVYIHPF-NH$_2$) Seplife C2 AM polyacrylate resin

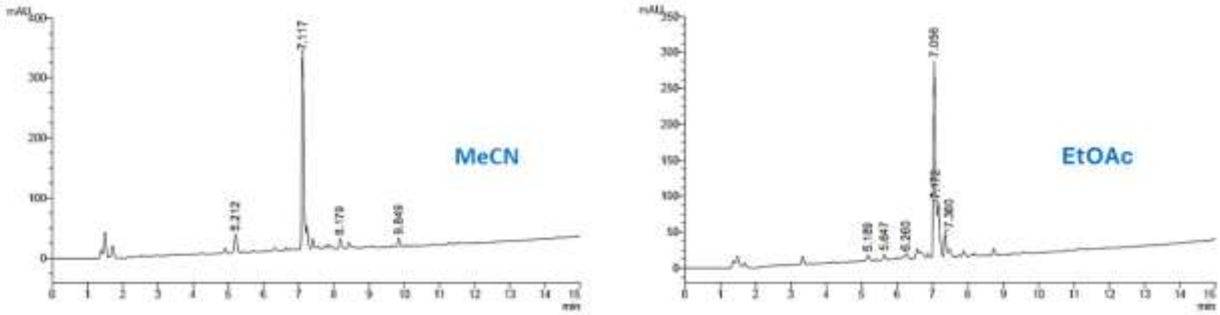

Detector A Channel 1 220nm

Peak#	Ret. Time	Height	Height%	Area	Area%
1	5.212	29928	7.604	147278	8.467
2	7.117	332858	84.571	1469779	84.500
3	8.179	16659	4.233	70770	4.069
4	9.849	14137	3.592	51558	2.964
Total		393582	100.000	1739384	100.000

Detector A Channel 1 220nm

Peak#	Ret. Time	Height	Height%	Area	Area%
1	5.189	8100	2.564	42584	4.159
2	5.647	6421	2.032	22400	2.188
3	6.260	4135	1.309	14608	1.427
4	7.056	237573	75.191	795141	77.653
5	7.172	29362	9.293	59099	5.772
6	7.360	30370	9.612	90009	8.791
Total		315962	100.000	1023840	100.000

Figure SI-19: HPLC for angiotensin at 45°C in MeCN and EtOAc.

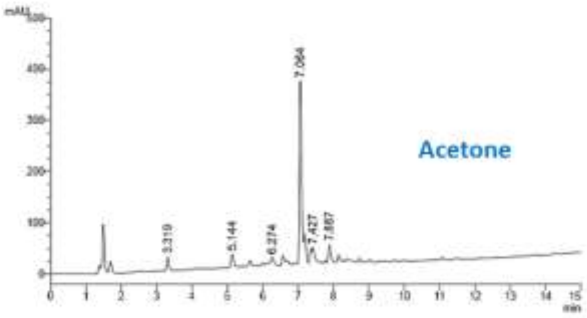

Detector A Channel 1 220nm

Peak#	Ret. Time	Height	Height%	Area	Area%
1	3.319	26446	5.514	113473	4.657
2	5.144	20596	4.294	83428	3.424
3	6.274	13369	2.787	73083	3.000
4	7.064	357385	74.510	1813463	74.432
5	7.427	28626	5.968	211317	8.673
6	7.887	33225	6.927	141651	5.814
Total		479646	100.000	2436414	100.000

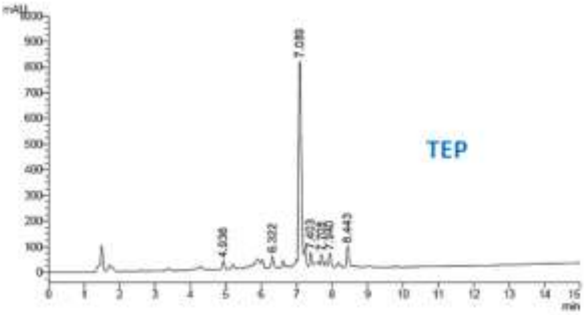

Detector A Channel 1 220nm

Peak#	Ret. Time	Height	Height%	Area	Area%
1	4.936	35395	3.340	146643	3.177
2	6.322	43790	4.132	189266	4.101
3	7.089	764842	72.170	3476310	75.315
4	7.403	51435	4.853	173722	3.764
5	7.708	36340	3.429	90171	1.954
6	7.940	48103	4.539	174671	3.784
7	8.443	79871	7.537	364905	7.906
Total		1059776	100.000	4615689	100.000

Figure SI-20: HPLC for angiotensin at 45°C in Acetone and TEP.

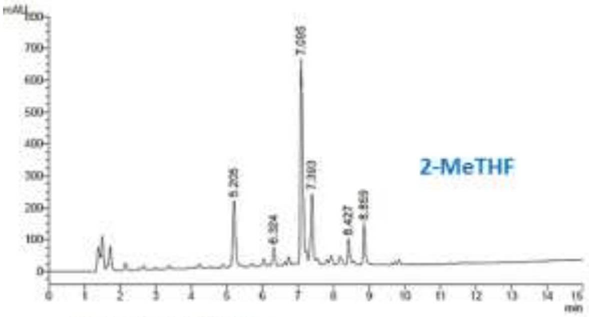

Detector A Channel 1 220nm

Peak#	Ret. Time	Height	Height%	Area	Area%
1	5.205	204398	15.842	1055017	18.315
2	6.324	55688	4.316	236119	4.099
3	7.095	626372	48.547	2890419	50.177
4	7.393	206197	15.981	854406	14.832
5	8.427	70921	5.497	211608	3.673
6	8.859	126650	9.816	512852	8.903
Total		1290225	100.000	5760421	100.000

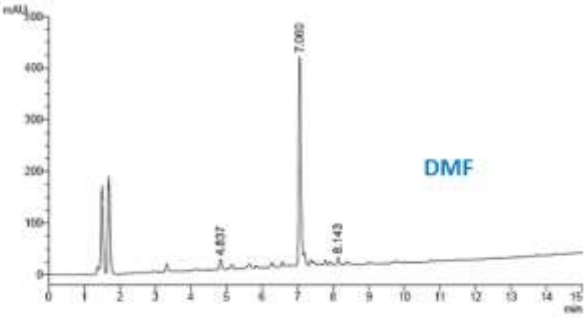

Detector A Channel 1 220nm

Peak#	Ret. Time	Height	Height%	Area	Area%
1	4.837	20265	4.614	89692	4.694
2	7.060	404491	92.094	1770391	92.648
3	8.143	14460	3.292	50792	2.658
Total		439216	100.000	1910875	100.000

Figure SI-21: HPLC for angiotensin at 45°C in 2-MeTHF and DMF.

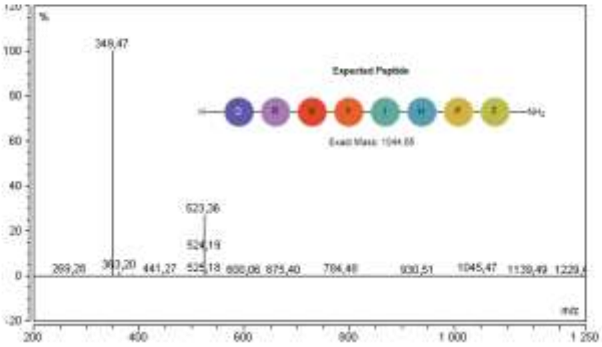

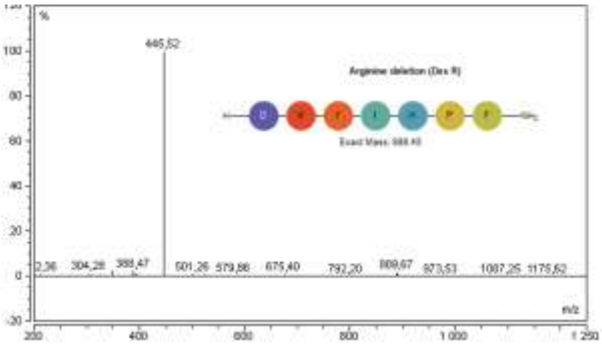

Figure SI-22: LCMS for angiotensin (DRVYIHPF) and Des R (DVYIHPF).

Synthesis of an analogue of Afamelanotide SYSLEHFRWGKPV-NH_2

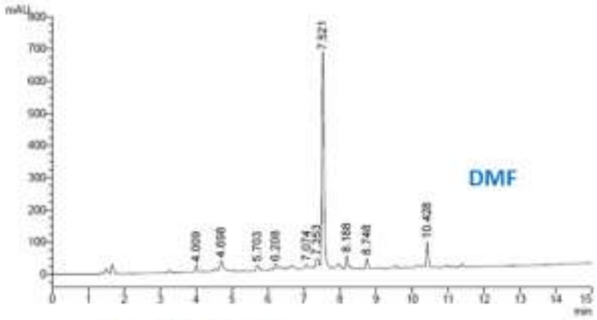

Detector A Channel 1 220nm

Peak#	Ret. Time	Height	Height%	Area	Area%
1	4.009	21916	2.369	84432	2.186
2	4.696	29192	3.156	160317	4.151
3	5.703	16856	1.822	85040	2.202
4	6.208	14311	1.547	51250	1.327
5	7.074	11292	1.221	54776	1.418
6	7.353	25843	2.793	132027	3.418
7	7.521	662130	71.573	2768106	71.668
8	8.188	37183	4.019	143798	3.723
9	8.748	31377	3.392	118554	3.069
10	10.428	75019	8.109	264118	6.838
Total		925118	100.000	3862418	100.000

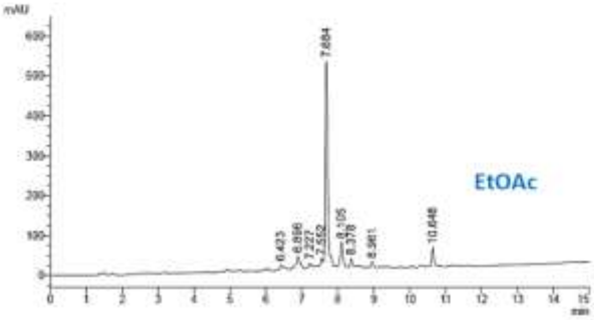

Detector A Channel 1 220nm

Peak#	Ret. Time	Height	Height%	Area	Area%
1	6.423	10161	1.455	40483	1.396
2	6.896	26650	3.817	154253	5.318
3	7.227	9762	1.398	42855	1.477
4	7.552	13714	1.964	42108	1.452
5	7.684	496959	71.178	2031293	70.028
6	8.105	60670	8.690	305055	10.517
7	8.378	20101	2.879	70639	2.435
8	8.961	14372	2.058	57187	1.971
9	10.648	45801	6.560	156834	5.407
Total		698191	100.000	2900707	100.000

Figure SI-23: HPLC for Afamelanotide SYSLEHFRWGKPV-NH_2 in DMF and EtOAc.

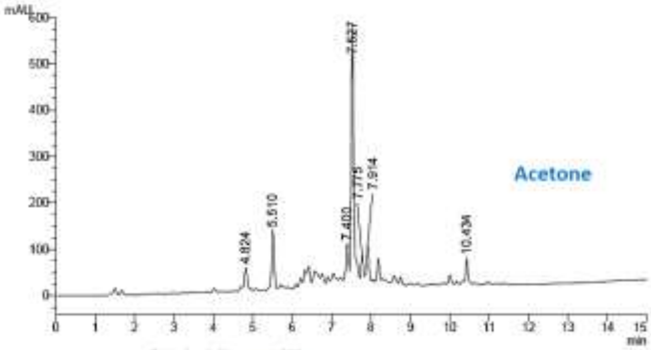

Detector A Channel 1 220nm

Peak#	Ret. Time	Height	Height%	Area	Area%
1	4.824	42254	4.614	186710	5.224
2	5.510	123117	13.444	533511	14.929
3	7.400	65637	7.167	258668	7.238
4	7.527	522032	57.003	1999243	55.943
5	7.775	44090	4.814	144588	4.046
6	7.914	67670	7.389	276102	7.726
7	10.434	51002	5.569	174910	4.894
Total		915801	100.000	3573731	100.000

Figure SI-24: HPLC for Afamelanotide SYSLEHFRWGKPV-NH_2 in Acetone.

Synthesis of GPSSGAPPPS-NH_2 on Seplife C2 AM polyacrylate resin

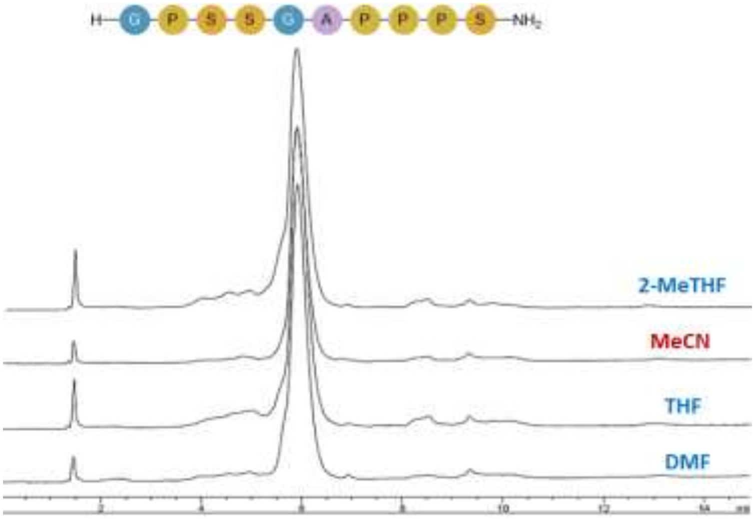

Figure SI-25: Synthesis of H-GPSSGAPPPS-NH_2 at 45°C. gradient: 0–30 % B into A in 15 min; flow rate: 1 mL/min; detection at 220 nm.

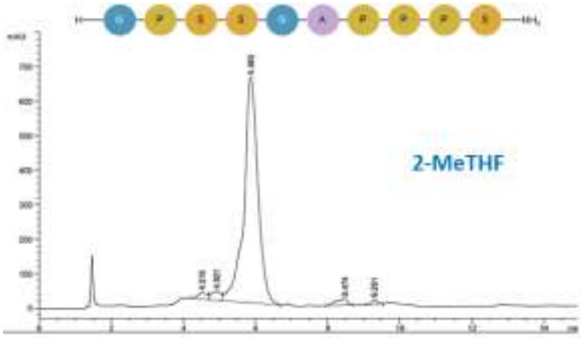

Peak #	RetTime [min]	Type	Width [min]	Area mAU *s	Height [mAU]	Area %
3	5.865	VB	0.3963	1.76606e4	654.53894	92.8829
4	8.474	BB	0.2902	401.27802	17.93161	2.1105
5	9.291	BB	0.1512	146.47597	14.16981	0.7704

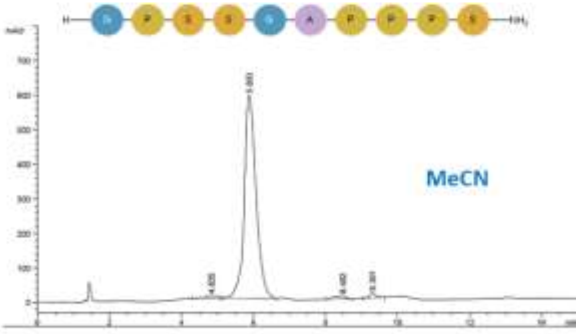

Peak #	RetTime [min]	Type	Width [min]	Area mAU *s	Height [mAU]	Area %
1	4.835	BB	0.2740	181.33212	8.10854	1.3074
2	5.883	BB	0.3467	1.32827e4	587.57703	95.7695

Figure SI-26: HPLC for H-GPSSGAPPPS-NH_2 in 2-MeTHF and MeCN.

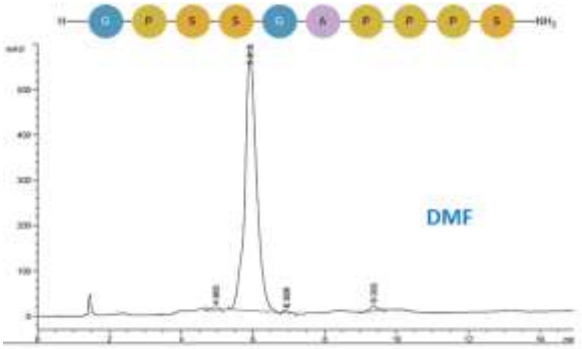

Peak #	RetTime [min]	Type	Width [min]	Area mAU *s	Height [mAU]	Area %
1	4.965	VB	0.2196	106.45495	5.97228	0.8021
2	5.916	BB	0.3409	1.29321e4	565.75366	97.4432

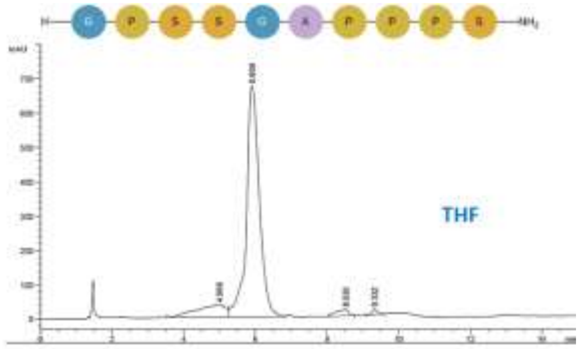

Peak #	RetTime [min]	Type	Width [min]	Area mAU *s	Height [mAU]	Area %
1	4.999	BV	0.6938	2132.35010	36.78077	10.8127
2	5.909	VV	0.3807	1.69750e4	475.05811	86.0771

Figure SI-27: HPLC for H-GPSSGAPPPS-NH_2 in DMF and THF.

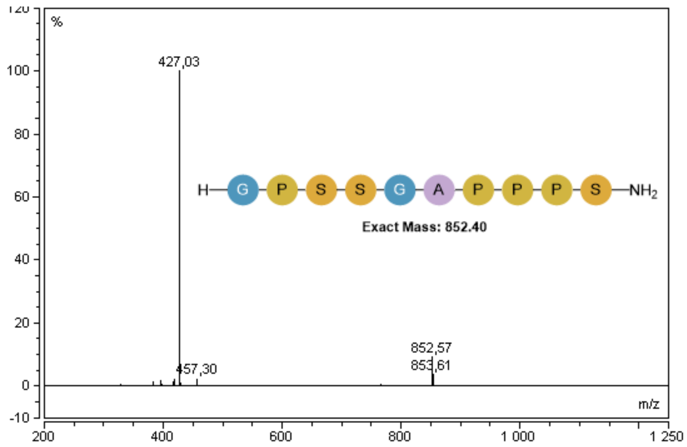

Figure SI-28: LCMS for H-GPSSGAPPPS-NH_2.

Synthesis of Fmoc-GPSSGAPPPS-NH_2 on Seplife C2 AM polyacrylate resin

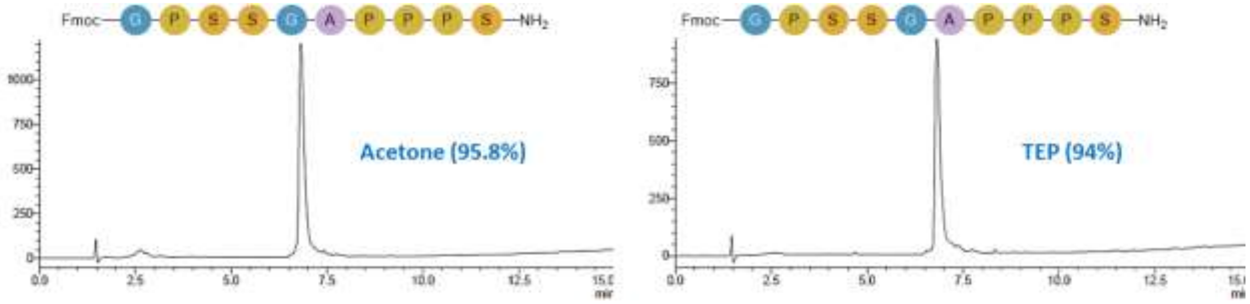

Figure SI-29: HPLC for Fmoc-GPSSGAPPPS-NH_2 in acetone and TEP

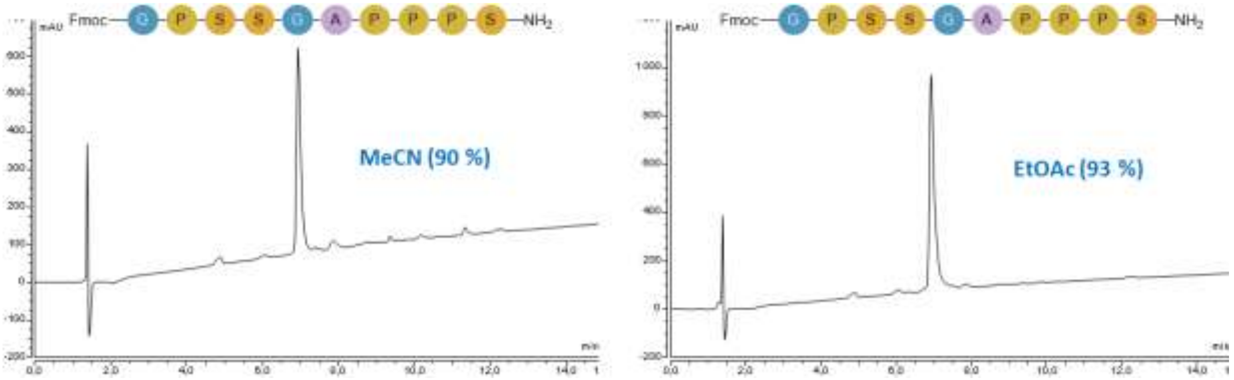

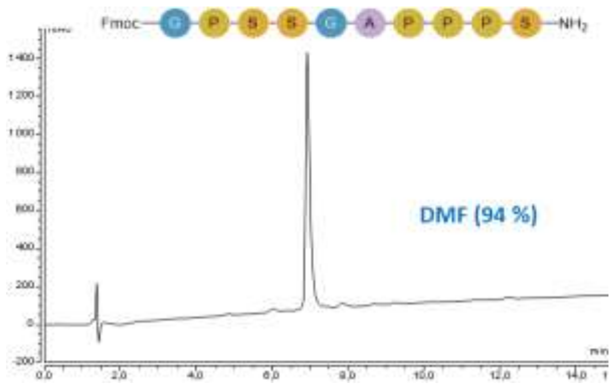

Figure SI-30: HPLC for Fmoc-GPSSGAPPPS-NH_2 in MeCN, EtOAc and DMF.

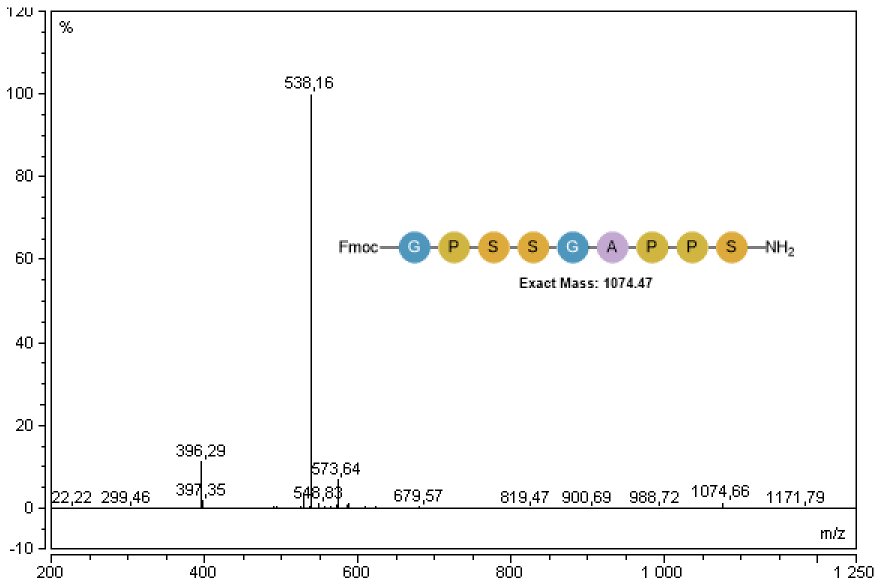

Figure SI-31: LCMS for Fmoc-GPSSGAPPPS-NH_2.
